# Green tea powder and *Lactobacillus plantarum* affect gut microbiota, lipid metabolism and inflammation in high-fat fed C57BL/6J mice

**DOI:** 10.1186/1743-7075-9-105

**Published:** 2012-11-26

**Authors:** Ulrika Axling, Crister Olsson, Jie Xu, Céline Fernandez, Sara Larsson, Kristoffer Ström, Siv Ahrné, Cecilia Holm, Göran Molin, Karin Berger

**Affiliations:** 1 Department of Experimental Medical Science, Lund University, Lund, Sweden; 2 Department of Applied Nutrition and Food Chemistry, Section of Food Hygiene, Lund University, Lund, Sweden; 3 Present address: Department of Health Sciences, Swedish Winter Sports Research Centre, Mid Sweden University, Östersund, Sweden

**Keywords:** Green tea, *Lactobacillus plantarum*, Type 2 diabetes, Obesity, Microbiota, *Akkermansia*, Inflammation, C57BL/6J

## Abstract

**Background:**

Type 2 diabetes is associated with obesity, ectopic lipid accumulation and low-grade inflammation. A dysfunctional gut microbiota has been suggested to participate in the pathogenesis of the disease. Green tea is rich in polyphenols and has previously been shown to exert beneficial metabolic effects. *Lactobacillus plantarum* has the ability to metabolize phenolic acids. The health promoting effect of whole green tea powder as a prebiotic compound has not been thoroughly investigated previously.

**Methods:**

C57BL/6J mice were fed a high-fat diet with or without a supplement of 4% green tea powder (GT), and offered drinking water supplemented with *Lactobacillus plantarum* DSM 15313 (Lp) or the combination of both (Lp + GT) for 22 weeks. Parameters related to obesity, glucose tolerance, lipid metabolism, hepatic steatosis and inflammation were examined. Small intestinal tissue and caecal content were collected for bacterial analysis.

**Results:**

Mice in the Lp + GT group had significantly more *Lactobacillus* and higher diversity of bacteria in the intestine compared to both mice in the control and the GT group. Green tea strongly reduced the body fat content and hepatic triacylglycerol and cholesterol accumulation. The reduction was negatively correlated to the amount of *Akkermansia* and/or the total amount of bacteria in the small intestine. Markers of inflammation were reduced in the Lp + GT group compared to control. PLS analysis of correlations between the microbiota and the metabolic variables of the individual mice showed that relatively few components of the microbiota had high impact on the correlation model.

**Conclusions:**

Green tea powder in combination with a single strain of *Lactobacillus plantarum* was able to promote growth of *Lactobacillus* in the intestine and to attenuate high fat diet-induced inflammation. In addition, a component of the microbiota, *Akkermansia*, correlated negatively with several metabolic parameters known to be risk factors for the development of type 2 diabetes.

## Background

The prevalence of obesity and associated type 2 diabetes is increasing rapidly and is a worldwide health problem [1]. The mechanisms whereby obesity leads to insulin resistance and type 2 diabetes involve ectopic lipid accumulation and low-grade inflammation. Non-alcoholic fatty liver disease is strongly associated with both obesity and type 2 diabetes [2]. Also, the pathogenesis of obesity and type 2 diabetes has been associated with a dysfunctional gut microbiota [3,4]. The mammalian gut is colonised with a diverse range of microorganisms that are known to play an important role in host metabolism [3,5,6]. Manipulation of the microbiota in mice using prebiotics [7] or antibiotics [8,9] has been shown to have beneficial effects on adiposity, glucose tolerance and inflammation. Also, addition of a probiotic mixture of strains has been shown to improve high-fat diet-induced hepatic steatosis, inflammation and insulin sensitivity in rodents [10,11]. Thus, the composition of the gut microbiota seems to play an important role in the regulation of lipid and glucose metabolism, as well as in inflammatory pathways.

Most studies on the microbiota in animals and humans have focused on caecum (rodents), colon (humans) or faeces as a reflection of the caecal or colonic microbiota. However, the small intestine appears to play an important role in the development of obesity and type 2 diabetes, as illustrated by the outcome of bariatric surgery where by-passing parts of the upper human gastrointestinal tract promotes weight-loss, ameliorate type 2 diabetes and improves hyperlipidemia [12].

Green tea is suggested to possess health promoting properties. It is a rich source of polyphenols, especially catechins, which represent approximately 30% of fresh leaf dry weight [13]. Extract from green tea or tea catechins have previously been shown to have anti-obesity and anti-diabetic effects in animal models [14-19] as well as in humans [20-22]. Most studies regarding health effects of green tea have been performed on tea extract or catechin fractions. Few studies have dealt with the effects of powdered whole green tea leafs, which include both water- and non-water soluble polyphenols as well as dietary fibres. Green tea powder is increasingly included as a supplementary ingredient in foods, e.g. in bakeries and desserts. Green tea catechins are poorly absorbed in the intestine and are thus present in the intestinal lumen where they are suggested to inhibit lipid absorption by forming complexes with lipids and lipolytic enzymes [23].

The species *Lactobacillus plantarum* (*L. plantarum*) has the ability to metabolize phenolic acids [24] and to split up tannins [25,26]. The metabolites are presumably more easily absorbed and distributed into the tissues where they can act as antioxidants and electron scavengers. Phenolic compounds can also have antimicrobial effects that may affect the composition of the gut microbiota, in favour of polyphenol-metabolizing components of the microbiota [27]. Also, green tea extracts have been shown to selectively inhibit the growth of pathogenic bacteria while either enhancing or not affecting the growth of beneficial bacteria like lactic acid bacteria [28-30]. To the best of our knowledge, the impact of green tea powder as a prebiotic compound to promote lactobacilli or other health promoting components of the microbiota has not previously been evaluated.

We report here a comprehensive study on the effects of green tea powder, with or without the addition of *L. plantarum* DSM 15313, on metabolic parameters and on the microbiota in both the small intestine and in the caecum of high-fat fed C57BL/6J mice. Our results demonstrate that the combination of green tea powder and *L. plantarum* alter the intestinal microbiota, reduce inflammation and affect cholesterol metabolism in a mouse model for human obesity and insulin resistance.

## Methods

### Animals and study design

The study followed the European Community regulations for animal experiments and was approved by the local animal ethics committee, Lund, Sweden, (permission number M202-08). Eight-week old female C57BL/6JBomTac mice, weighing 18.2 ± 2.2 g (Taconic, Skensved, Denmark) were housed 7 mice/cage in a controlled environment (12 hour light cycle). After one week of acclimatization, the mice were randomly divided into four groups (n = 21), and thereafter fed the experimental diets *ad libitum* with free access to drinking water. The two experimental diets were high-fat diets (HFD, 45 energy% fat; Research Diets, NB, USA), with (D08021404) or without (D12451N) supplement of 4% (w/w) powdered green tea leaves (*Camellia sinensis,* Premium Powdered Sencha, http://www.JapaneseGreenTeaOnline.com). The green tea powder was analyzed for nutritional content including water soluble and insoluble fibers (Eurofins, Lidköping, Sweden) as well as water soluble and insoluble polyphenols, see Additional files 1 and 2, respectively. The drinking water was supplemented with 1.5% (v/v) of either freezing medium (3.6 mM K_2_HPO_4_, 1.3 mM KH_2_PO_4_, 2.0 mM sodium citrate, 1.0 mM MgSO_4_, 12% glycerol) or *L. plantarum* DSM 15313 (strain HEAL19, Probi AB, Lund, Sweden) suspended in freezing medium to a concentration of 3 × 10^9^ cfu/ml drinking water, giving an approximate intake of 1 × 10^10^ cfu/day. The concentrated aliquots of bacteria suspension were stored in −20°C and mixed with fresh tap water on a daily basis. *L. plantarum* has previously been shown to be stable during 24 hours in tap water at room temperature [31]. Food intake (on a per cage basis) and body weights were registered once a week. After 5, 11 and 22 weeks, body fat mass was analyzed using dual-energy X-ray absorptiometry technique with a Lunar PIXImus densitometer (GE Medical Systems). Oral glucose tolerance tests were performed after 8 and 21 weeks and an intravenous insulin tolerance test was performed after 15 weeks. Two mice in the Lp group did not wake up from the anaesthesia after the first oral glucose tolerance test. Faeces were collected at three different time points during the study. Mice were placed in clean cages with a minimum of bedding for 24 hours. Faeces from each cage was collected, lyophilized and grounded in a mortar and stored at −20°C until analysis. After 11 weeks, 10 mice in each group were sacrificed in order to study time dependency of the treatment, and after 22 weeks, the remaining mice (9–11 per group) were sacrificed. At the time of sacrifice, mice were fasted for 4 hours and thereafter anesthetized with an intraperitoneal injection of midazolam (Dormicum, Hoffman- La Roche, Basel, Switzerland) and a mixture of fluanison/fentanyl (Hypnorm, Janssen, Beerse, Belgium). Blood was drawn by intraorbital puncture followed by cervical dislocation. Periovarian white adipose tissue, liver (rinsed with PBS) and spleen were weighed and snap-frozen in liquid nitrogen. A part of the duodenum (3–10 mm distally of pylorus) was collected in buffer containing 10 mM Tris-HCl and 1 mM EDTA, pH 8.0, and snap-frozen in liquid nitrogen. The caecum including its content was weighed and thereafter the wall was removed. Parts of the content were frozen in freezing medium for culture of bacteria and parts were snap frozen for extraction of bacterial DNA. All samples were stored at −80°C until analysis.

### Viable count

Plate count was performed on the caecum content and the samples were cultivated on Violet Red Bile Dextrose agar (*Enterobacteriaceae*) incubated aerobically at 37°C for 24 hours and on Rogosa agar (lactobacilli) incubated anaerobically at 37°C for 48 hours. From Rogosa plates, 1–2 colonies morphologically resembling *L. plantarum* were picked for tentative identification of *L. plantarum* DSM 15313 by Random Amplified Polymorphic DNA (RAPD) analysis (for details regarding RAPD see Additional file 3).

### DNA extraction from intestinal samples

DNA was extracted from the caecum content using a Biorobot EZ1 and a DNA Tissue Kit (Qiagen AB, Sollentuna, Sweden) as previously described [32]. Extracted DNA was eluted in 200 μl by the Biorobot EZ1 according to the manufacturer’s instruction.

### Amplification and T-RFLP analysis

The universal primers ENV1 and ENV2 were used for amplification of the bacterial 16S rRNA genes [33]. The forward ENV1 primer was fluorescently labelled with FAM. The amplicons were then purified and digested with the restriction endonuclease *Msp*I. For caecum samples the restriction endonuclease *Alu*I (Fermenta Life Science) was also used (for details see Additional file 3). Samples were analyzed at DNA-lab (SUS, Malmö, Sweden). The total area was summarized for each sample and the relative area for each T-RF was calculated and expressed as a percentage of the total area. In order to identify peaks in the T-RFLP profile a pure culture of *L. plantarum* DSM 15313 and a selected clone obtained previously from mouse caecum content that had been identified by partial sequencing of the 16S rRNA genes as *Akkermansia* (unpublished results) were digested by *Msp*I and analyzed by T-RFLP.

### Cloning and sequencing of the 16S rRNA genes

The bacterial 16S rRNA genes of the small intestinal samples were amplified with the primers ENV1 and ENV2. The cloning was performed using the pGEM-T vector system II (Promega, Madison, USA) as previously described [33]. Randomly selected clones were harvested and stored in freezing media at −80°C (for details regarding cloning see Additional file 3). Clones were sent to MWG-Biotech (MWG-Biotech, Ebersberg, Germany) for sequencing using the primer Univ-0519-a-A-18 GWA TTA CCG CGG CKG CTG [34]. Approximately the first 450 bases were sequenced and the obtained sequences of 16S rRNA genes were checked and edited manually using Bioedit Sequence Alignment Editor Version 7.0.9.0 [35]. The sequences were compared with the Ribosomal Database Project, release 10 using the option “Sequence Match” (Ribosomal Database Project, http://rdp.cme.msu.edu/) [36]. Clone sequences were also compared by using the option “similarity table”, found at http://mobyle.pasteur.fr/cgi-bin/portal.py#forms::dnadist. Ten clones were selected, one from each operational taxonomic unit containing sequences showing >98% similarity, and submitted to GenBank. The accession numbers are JX971027-JX971036. Sequences are named with the acronym Msi (mice small intestine).

### SYBR Green quantitative PCR of bacteria in small intestine

Quantitative PCR (qPCR) was run separately in a Mastercycler® ep realplex 1.5 real-time PCR system (Eppendorf) using primers for *Lactobacillus*, *Akkermansia*, *Enterobacteriaceae* and total bacteria (for primer sequences and details see Additional files 3 and 4). The standards for qPCR were prepared by cloning the target fragments of the 16S rRNA genes from *L. plantarum* CCUG 35035 and *E. coli* CCUG 29300 (Culture collection, University of Gothenburg, Sweden). The former was used as template for *Lactobacillus* and total bacteria and the latter for *Enterobacteriaceae*. For *Akkermansia* a previously obtained clone from mouse caecum was used for the preparation of a standard (for details see Additional file 3).

### Diversity index

For comparison of the microbial diversity between the groups both the Shannon and Simpson’s diversity indices were calculated [37,38]. Both indices take evenness and species richness into account but the Simpson index is more weighted towards the abundance of the most common species than species richness [38]. The area of each T-RF, expressed as the proportion of the total area for a sample, was used for calculation.

### Lipid extraction from faeces and liver

Lipids in faeces were extracted with hexane:isopropanol (3:2) and dried under nitrogen. The lipids were re-dissolved in isopropanol. Whole liver was grounded to a homogenous powder in a mortar in liquid nitrogen. An aliquot of the powdered liver was homogenized in phosphate buffered saline and thereafter lipids were extracted with chloroform:methanol (2:1) overnight. The organic phase was washed, dried under nitrogen and re-suspended in chloroform. Triacylglycerol (TAG) and cholesterol were analyzed (Infinity, Thermo Electron Melbourne, Australia).

### RNA preparation from liver tissue and real-time qPCR

Total RNA was isolated from grounded liver (see above) using the RNeasy Mini Plus Kit (Qiagen, Hilden, Germany) according to the manufacturer’s instruction. Total RNA (1 μg) was treated with DNaseI amplification grade (Invitrogen, Carlsbad, CA, USA) and reversely transcribed using random hexamers (Amersham Bioscieces, Piscataway, NJ, USA) and SuperScript II RNaseH reverse transcriptase (Invitrogen) according to the manufacturer’s recommendations. The cDNA was used for quantitative PCR using TaqMan chemistry (assays-on-demand, Applied Biosystems) or SYBR green chemistry (primers were designed using the software Primer Express, Applied Biosystems), with an ABI 7900 system (Applied Biosystem, Foster City, CA, USA), see Additional file 5 for assay IDs of the Taqman probes and list of primer sequences. The relative quantification of mRNA was calculated using the ΔΔCt-method with normalization by geometric average of the two housekeeping genes ribosomal protein S29 (RPS29) and glyceraldehyde-3-phosphate dehydrogenase (GAPDH) [39].

### Oral glucose tolerance test

Mice fasted for 10 hours (9 p.m.–7 a.m.) were anesthetized with 0.4–0.6 mg Fluanison, 0.01–0.02 mg Fentanyl (Hypnorm) and 0.2–0.3 mg Midazolam (Dormicum) per mouse. D-glucose (75 mg in 0.5 ml) was given by intragastric gavage. Blood samples were drawn by intraorbital puncture using an EDTA-coated glass pipette at 0, 15, 30, 60 and 120 min after glucose administration. After immediate centrifugation plasma was collected and stored at −20°C until analysis of glucose and insulin.

### Insulin tolerance test

Mice fasted for 4 hours (9 a.m.–1 p.m.) were anesthetized as above. Insulin (Actrapid®, Novo Nordisk A/S, Denmark) was injected intraperitoneally (0.75 mU/g body weight). Blood samples were drawn as above at 0, 15, 30, 45, 60 and 90 minutes after insulin administration for glucose analysis.

### HOMA index

Insulin resistance was assessed by the homeostasis model assessment (HOMA), a mathematical model describing the degree of insulin resistance from fasting glycaemia and insulineamia. HOMA-IR is calculated by multiplying fasting plasma insulin (mU/l) with fasting plasma glucose (mmol/l) divided by 22.5 [40].

### Plasma samples

At week 11 and 22 blood was drawn from 4 hour fasted anesthetized mice by intraorbital puncture. Plasma was prepared and stored as above. Glucose, cholesterol, TAG (Infinity, Thermo Electron Melbourne, Australia), non-esterified fatty acids (NEFA) (NEFA C, Wako Chemicals, Neuss, Germany), fructosamine (VetSpec^TM^, Catachem Inc., Oxford, CT, USA) and alanine aminotransferase (ALT) (DiscretePak^TM^, Catachem Inc.) were measured enzymatically. Insulin and adiponectin were measured radioimmunochemically (Linco Research, St Charles, MO, USA). Leptin, interleukin-6 (IL-6), monocyte chemoattractant protein −1 (MCP-1) and plasminogen activator inhibitor-1 (PAI-1) were analyzed in an aliquot of plasma that had been snap-frozen in liquid nitrogen and stored at −80°C using Luminex technology (LX200, Luminex Corporation, Austin, TX, USA). Before the onset of the study 10 mice were sacrificed and blood was collected to measure baseline glucose and insulin.

### Statistics

For the bacterial analyses, multiple groups’ comparisons were performed using Kruskal-Wallis test, pair-wise comparisons using Nemenyi-Damico-Wolfe-Dunn test (NDWD), and correlations using Spearman’s rank correlation test with Bonferroni correction for multiple testing. Correlation tests were carried out between the total amount of bacteria in the small intestine, amount of *Lactobacillus* and *Akkermansia* as well as small intestinal and caecal diversity, and body weight, total body fat content, periovarian white adipose tissue, plasma leptin, plasma fructosamine, basal plasma glucose and insulin, the area under the curve for the oral glucose tolerance tests, liver weight, hepatic TAG content, plasma ALT, plasma TAG, plasma NEFA, plasma adiponectin, plasma PAI-1, spleen weight and caecum weight. The statistical tests were performed using the stat package [41,42] and the add-on package “coin” [43,44] in the statistical software R (version 2.10.1, R Development Core Team, 2009).

For the rest of the data, one-way analysis of variance (ANOVA) with Bonferroni’s multiple comparison post-test was used when the data was Gaussian distributed according to D’Agostino and Pearson omnibus normality test. When not normally distributed, Kruskal-Wallis non-parametric test with Dunn’s multiple comparison post-test was used. Also, two-way ANOVA with Bonferroni post-test was used to compare body weights. The statistical analyses were performed with the GraphPad Software (GraphPad Prism 5.0, San Diego, CA, USA). All tests were two-sided and data were considered significant if *P* < 0.05.

### Multivariate data analysis

Principal component analysis (PCA) was applied to the T-RFLP data (a matrix of the relative peak area) and to the metabolic data in order to clarify cluster-structures in the data sets (SIMCA-P software, version 12.0.1; Umetrics, Umeå, Sweden). The two sets of data were used to build a Partial Least Squares Projections to Latent Structures (PLS) model in order to find correlations between them (Autofit in the SIMCA-P). First, all the metabolic parameters were used to build the PLS model, thereafter, those with Q2 < 0.2, which indicates a weak correlation against the small intestinal or caecal T-RFLP pattern, were excluded from the model.

## Results

### Microbiota of the small intestine

**Figure 1:**
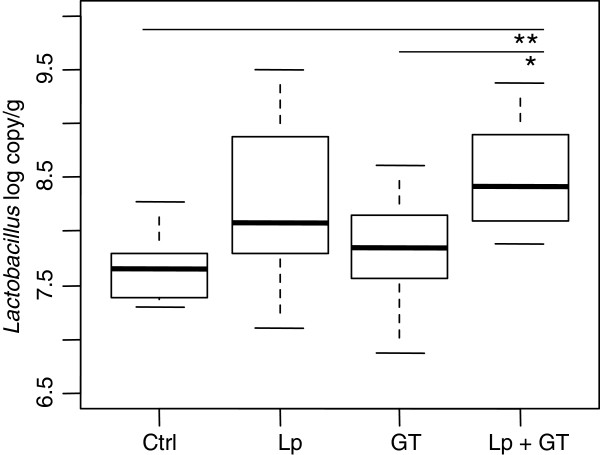
**Quantification (PCR-copies) of *Lactobacillus *in small intestinal tissue after 22 weeks.** Significant differences were reached for *Lactobacillus* between the control group and the Lp + GT group (p = 0.002), and between the groups receiving GT and Lp + GT (p = 0.04). Ctrl = high fat control diet (HFD), Lp = HFD + *L. plantarum* in the drinking water, GT = HFD supplemented with 4% green tea powder, Lp + GT = HFD supplemented with 4% green tea powder and *L. plantarum* in the drinking water.

The microbiota of the small intestine was analyzed by qPCR and T-RFLP at 22 weeks. Two samples were also subjected to cloning and sequencing of the 16S rRNA genes. The PCR-quantified load of *Lactobacillus* differed between the groups after 22 weeks (p = 0.002). Pair wise comparison showed that the Lp + GT group had significantly more *Lactobacillus* than the control group (p = 0.002) and the GT group (p = 0.04) (Figure 1). No differences in the amount of the Gram-negative and mucin degrading genus *Akkermansia* (belonging to the phylum *Verrucomicrobia*) or total amount of bacteria were observed between the groups. The mean values of log copies/g for *Akkermansia* were 6.75 ± 0.27 (control), 6.85 ± 0.75 (Lp), 6.97 ± 0.52 (GT) and 7.09 ± 0.43 (Lp + GT). For the total bacteria the mean values of bacterial copies were 8.66 ± 0.38 (control), 8.81 ± 0.53 (Lp), 9.02 ± 0.40 (GT), and 9.09 ± 0.38 (Lp + GT) log copies/g. No *Enterobacteriaceae* could be detected, except for in one individual (data not shown).

**Figure 2:**
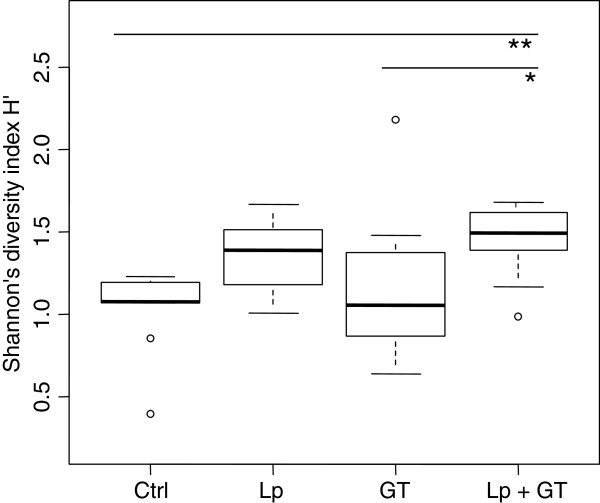
**Bacterial diversity in the small intestine after 22 weeks.** The data is based on T-RFLP-profiles and *Msp*1 digestion. The area of each peak, expressed as the proportion of the total area, was used for calculation of the Shannon diversity index. Control vs. Lp + GT (p = 0.008), GT vs. Lp + GT (p = 0.04).

T-RFLP was used for analyzing the diversity of the gut microbiota. When using restriction endonuclease *Msp*I the number of T-RFs varied between the animals and there was a significant difference in diversity based on the number of T-RFs among the four groups (p < 0.001). The median values of the peak numbers of the groups were 3.5, 6.0, 6.5 and 8.0 for control, Lp, GT and Lp + GT respectively. Pair wise comparison of the groups revealed significant differences between control and GT (p < 0.05), and between control and Lp + GT (p < 0.001). Shannon (H') as well as Simpsons’s diversity index (D) showed a significant difference among the four groups (p = 0.007 and p = 0.006 respectively). Pair-wise comparison showed that the Lp + GT group had a significantly more diverse microbiota than the control group (p = 0.008) and the GT group according to the Shannon diversity index (p = 0.04) (Figure 2). According to the Simpson’s diversity index the Lp group had a significantly higher diversity than the GT group (p = 0.04) (data not shown). A T-RF corresponding to *L. plantarum* DSM 15313 was found in 5 individuals in the Lp group and in 4 individuals in the Lp + GT group. In order to identify some of the T-RFs in the T-RFLP profiles, the two mice with the highest number of T-RFs in the control and Lp + GT group were selected for bacterial cloning. The obtained sequences were edited and 55 sequences were analyzed and compared to the closest matches in the RDP database [36] (Table 1). Fiftyone out of fiftyfive clones belonged to the phylum *Firmicutes.* Within *Firmicutes,* sequences similar to the family *Erysipelotrichaceae* were dominating. These sequences were most similar to the genus *Allobaculum*, but the similarity was only 89%. In the mouse from the Lp + GT group, 25.9% of the clones were identified as *Lactobacillus* compared to only 3.6% from the control mouse. Clones identified as *Lactobacillus* were most similar to sequences belonging to *Lactobacillus reuteri* and *Lactobacillus intestinalis*. Due to differences between the theoretical and the actual sizes of T-RFs of the clones no attempt was made to identify the T-RFs except for *Akkermansia* and the given *L. plantarum* strain which showed fragments length of 265 and 568 bp, respectively. A comparison was made between T-RFLP and the numbers of copies in the qPCR. The mean copy value for *Akkermansia* obtained in the qPCR for the T-RFs detected in the T-RFLP was log 7.28 ± 0.52 compared to a mean of log 6.75 ± 0.27 for copy number that was not detected by T-RFLP. No microbial analyses of the small intestine were performed at 11 weeks.

**Table 1:** Direct identification of 16S rRNA genes from small intestinal tissue by PCR-amplification, cloning and sequencing

**Phylum**	**Accession no.**	**Closest type strain**	**Group (No of clones)**^**a**^	**Similarity (%)**^**b**^
*Firmicutes*				
	X76329	*Lactobacillus pontis* (T)	Ctrl (1)	95.5
	AJ306299	*Lactobacillus intestinalis* (T)	Lp + GT (3)	99.2–99.5
	L23507	*Lactobacillus reuteri* (T)	Lp + GT (3)	95.3–95.7
	X94229	*Lactobacillus oris* (T)	Lp + GT (1)	95.1
	M59230	*Eubacterium biforme* (T)	Lp + GT (1)	89.5
	AJ417075	*Allobaculum stercoricanis* (T)	Ctrl (24) Lp + GT (17)	89.1 ~ 89.4
	X73447	*Clostridium irregulare* (T)	Ctrl (1)	96.1
*Verrucomicrobia*				
	AY271254	*Akkermansia muciniphila* (T)	Ctrl (1)	99.7
*Proteobacteria*				
	DQ422859	*Desulfovibrio bizertensis* (T)	Lp + GT (1)	88.8
*Actinobacteria*				
	AF292373	*Olsenella uli* (T)	Lp + GT (1)	94.4
	AB042288	*Propionibacterium acnes* (T)	Ctrl (1)	100

^a^Ctrl = high fat control diet (HFD), Lp + GT + HFD supplemented with 4% green tea powder and *L. plantarum* in the drinking water. Numbers within parentheses denote the number of clones in each mouse. ^b^Denotes highest sequence similarity to a type strain (T) found in the Ribosomal data base project Release 10 (http://rdp.cme.msu.edu/).

### Caecum weight and microbiota

After 11 weeks, the caecum weights were significantly higher in mice from the GT groups compared to mice in the control and Lp groups (control: 0.126 ± 0.006, Lp: 0.133 ± 0.007 GT: 0.225 ± 0.015, Lp + GT: 0.229 ± 0.015 g). After 22 weeks, the difference in caecum weights was even more pronounced (control: 0.125 ± 0.013, Lp: 0.148 ± 0.008, GT: 0.291 ± 0.027, Lp + GT: 0.332 ± 0.022 g). The caecum microbiota was analyzed by cultivation and by T-RFLP after 11 and 22 weeks. The viable count of lactobacilli in the caecum content was significantly higher in the Lp group (p <0.01) than in the control group at 11 weeks while the Lp + GT group had significant more lactobacilli than control (p < 0.01) and the GT-group (p < 0.05) after 22 weeks (see Additional file 6). *L. plantarum* DSM 15313 was found at both 11 and 22 weeks in the two groups fed *L. plantarum*. *L. plantarum* could be reisolated from 77% and 100% of the mice in the Lp + GT and Lp groups, respectively (data not shown). No significant difference in the viable count of *Enterobacteriaceae* was seen after 11 weeks in the three treatment groups compared to the control. After 22 weeks, the viable count of *Enterobacteriaceae* had decreased below the detection limit in more than 40% of the animals and no statistical analysis was made (data not shown).

T-RFLP analysis of the caecum content generated in total 72 different T-RFs after 11 weeks and 82 T-RFs after 22 weeks in the T-RFLP-profile using *Msp*I. At 11 weeks the GT group had significantly more T-RFs than the control when using *Msp*I (p < 0.01). For *Alu*I digestions, both the GT group and the Lp + GT group had significantly more T-RFs than the control group (p < 0.05). After 22 weeks there were no differences compared to the control with either *Msp*I or *Alu*I (data not shown). Shannon and Simpson diversity indices were calculated for each sample using the relative T-RF area. After 11 weeks both the Shannon and the Simpson indices showed a significantly higher diversity of the microbiota in the GT group compared to the control when using *Alu*1 digestion for the former and either *Msp*I or *Alu*1 digestion for the later (see Additional file 7). After 22 weeks, no significant differences in the bacterial diversity of the caecum content could be seen between the treatment groups and the control (see Additional file 7). In order to perform a putative identification of T-RFs from the caecum content, a pure culture of *L. plantarum* DSM 15313 was analyzed by T-RFLP, showing a single T-RF of 568 base pairs. This T-RF was not found in the control group or in the GT group but in all animals of the Lp group and in 75% of the mice in the Lp + GT group. Also, a T-RF (265 bp) corresponding to *Akkermansia* was found in 27 mice of 29 after 11 weeks and in 40 mice of 43 after 22 weeks.

### Adiposity

**Figure 3:**
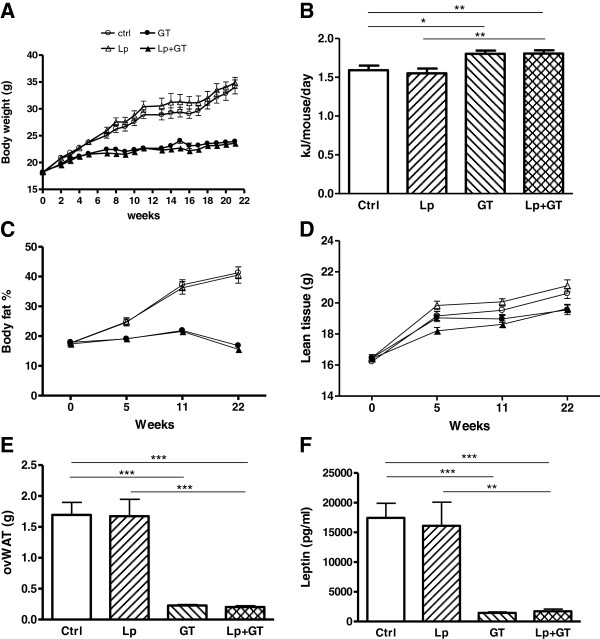
**Decreased adiposity in mice fed a diet supplemented with green tea.** (**A**) Weekly body weight registration during the 22 week study. (**B**) Average energy intake calculated as KJ/mouse/day. (**C**) Relative body fat content (%) recorded using DEXA scan technique at week 0, 5, 11 and 22. (**D**) Lean body mass measured with DEXA at 0, 5, 11 and 22 weeks. (**E**) Periovarian white adipose tissue weight and (**F**) plasma leptin concentration after 22 weeks on the different diets. Ctrl = high fat control diet (HFD), Lp = HFD + *L. plantarum* in the drinking water, GT = HFD supplemented with 4% green tea powder, Lp + GT = HFD supplemented with 4% green tea powder and *L. plantarum* in the drinking water. Data are means ± SEM n = 21 at 11 weeks and n = 9–11 at 22 weeks. *p < 0.05, **p < 0.01, ***p < 0.001.

Body weight gain and relative body fat content was significantly reduced in mice of the GT groups compared to control mice (Figure 3A, C). A supplement of Lp had no significant effect on body weight or body fat content. The mean energy intake was higher in mice fed GT compared to mice fed a diet without GT (Figure 3B). In addition, periovarian white adipose tissue depots (Figure 3E) and circulating leptin (Figure 3F) were strongly reduced in mice receiving GT. No significant difference in lean body mass was observed between groups (Figure 3D). The amount of *Akkermansia* in the small intestine correlated negatively with body fat content (rho = −0.43; p = 0.04), periovarian white adipose tissue (rho = −0.43; p = 0.03) as well as plasma leptin (rho = −0.45; p = 0.03). The total amount of bacteria in the small intestine correlated negatively with periovarian white adipose tissue (rho = −0.41; p = 0.04) and showed a tendency towards a negative correlation with total body fat (rho = −0.41; p = 0.07) and plasma leptin (rho = −0.35; p = 0.09). The mean faecal TAG excretion was significantly elevated in the GT groups compared to control (control: 0.08 ± 0.01, Lp: 0.18 ± 0.05, GT: 0.27 ± 0.04, Lp + GT: 0.31 ± 0.04 mg/mouse/24 h). The total amount of faeces was not significantly different between the groups although a tendency towards increased excretion was observed in the GT groups compared to the control group (ctrl: 0.27 ± 0.02, Lp: 0.30 ± 0.04, GT: 0.39 ± 0.06, Lp + GT: 0.40 ± 0.06 mg/mouse/24 h).

### Blood glucose control

**Figure 4:**
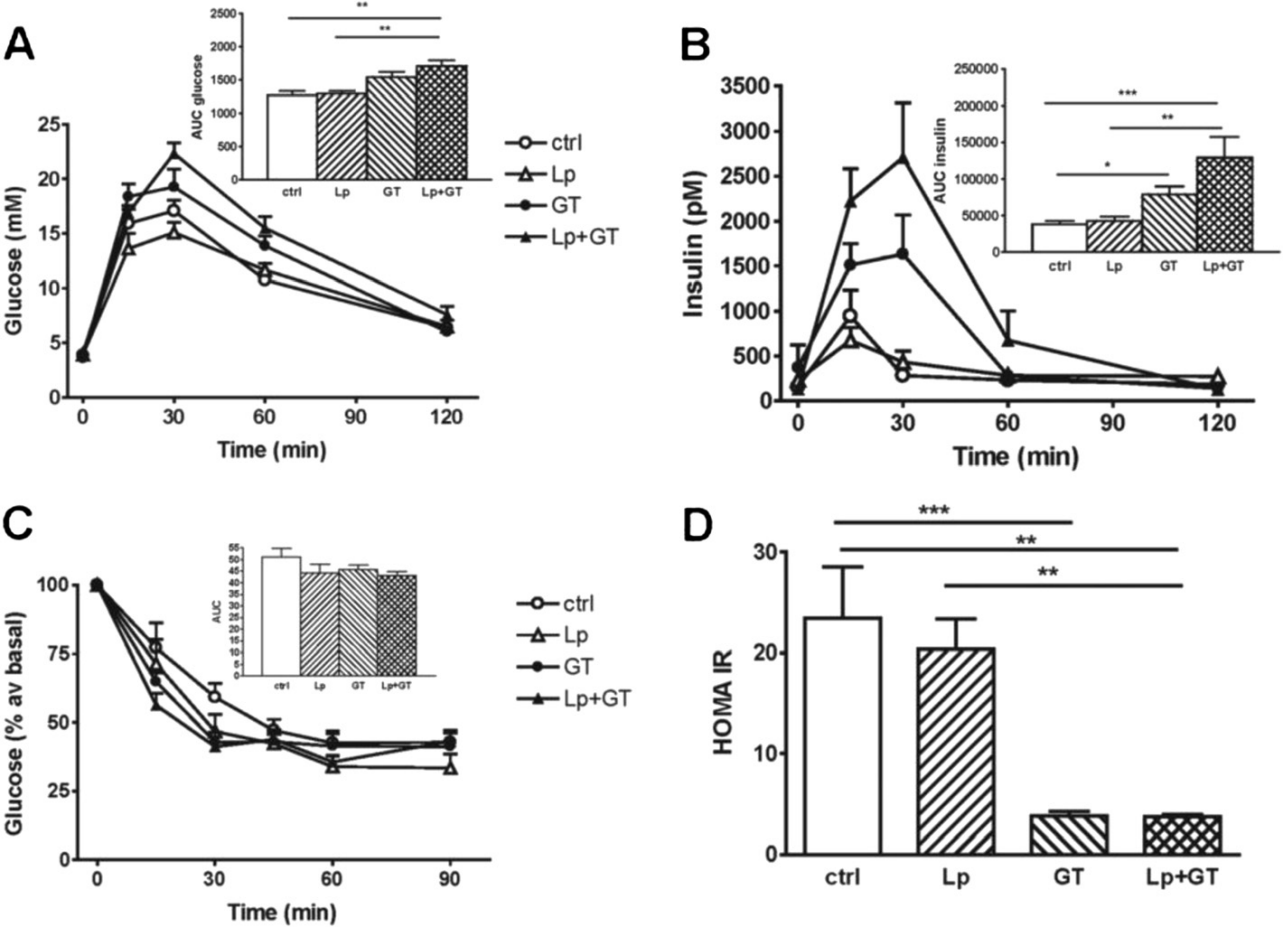
**Altered glucose and insulin tolerance by supplementing the diet with green tea.** (**A**) Plasma glucose and (**B**) insulin concentrations in an oral glucose tolerance test performed at week 8. Mean values and SEM for 11 mice in each group. (**C**) Plasma glucose expressed as % of basal after an intraperitoneal injection of insulin at week 15. Mean values and SEM for 9–11 mice in each group. No differences were detected between any of the four groups. The areas under the curves (AUC) are shown in the insets (**D**) HOMA-IR index at week 22. The index was calculated by multiplying fasting glucose (mM) and fasting insulin (μU/ml) divided by 22.5. Ctrl = high fat control diet (HFD), Lp = HFD + *L. plantarum* in the drinking water, GT = HFD supplemented with 4% green tea powder, Lp + GT = HFD supplemented with 4% green tea powder and *L. plantarum* in the drinking water. Data are means ± SEM for 9–11 mice/group. *p < 0.05, **p < 0.01, ***p < 0.001.

Oral glucose tolerance tests were performed at week 8 and week 21. At week 8, the mice in the two GT groups had a significantly increased insulin response compared to control mice (Figure 4B). Also, the Lp + GT group had a significantly increased glucose response compared to the control and the Lp group (Figure 4A). The same tendencies were seen at week 21, however, the differences did no longer reach statistical significance (Additional file 8). An insulin tolerance test was performed at week 15. No significant differences were observed between any of the groups (Figure 4C)*.* After 22 weeks, fasting plasma glucose, insulin and fructosamine were lower in the mice from the GT groups, while insulin and fructosamine were significantly lower already after 11 weeks (Table 2). In addition, after 22 weeks, GT resulted in a lower HOMA index of insulin resistance (Figure 4D). The amount of *Akkermansia* in the small intestine correlated negatively to plasma insulin (rho = −0.47; p = 0.03) and there was also a tendency towards a negative correlation between the total amount of bacteria and plasma insulin (rho-0.35; p = 0.07). No differences in plasma glucose or insulin were observed between the groups at the initiation of the study (data not shown).

**Table 2:** Plasma profile after indicated time of dietary treatment

	**Week**	**Ctrl**	**Lp**	**GT**	**Lp + GT**
Glucose (mM)	11	7.3 ± 0.4	8.2 ± 0.6	7.4 ± 0.7	7.5 ± 0.5
	22	9.3 ± 0.5*a#*	9.5 ± 0.7*a*	6.0 ± 0.4*b*	7.1 ± 0.3*b*
Insulin (pM)	11	289 ± 48*a*	403 ± 50*a*	177 ± 12*b*	169 ± 12*b*
	22	387 ± 72*a*	353 ± 58*a*	99 ± 6b*	100 ± 4b*
Fructosamine (μM)	11	535 ± 25*a*	629 ± 45*a*	429 ± 23*b*	545 ± 41b
	22	606 ± 18*a§*	582 ± 39*a*	521 ± 16*b#*	518 ± 21*b*
Cholesterol (mM)	11	4.2 ± 0.24*a*	4.3 ± 0.22	4.7 ± 0.22	4.8 ± 0.08*b*
	22	5.2 ± 0.3*a§*	5.0 ± 0.24*a*	3.9 ± 0.08b*	4.0 ± 0.18b*
TAG (mM)	11	0.41 ± 0.04a	0.42 ± 0.07	0.29 ± 0.03b	0.31 ± 0.02
	22	0.48 ± 0.05*a*	0.40 ± 0.05*a*	0.30 ± 0.02*b*	0.32 ± 0.03*b*
NEFA (mM)	11	0.57 ± 0.02*a*	0.59 ± 0.04*a*	0.47 ± 0.03*b*	0.52 ± 0.03*ab*
	22	0.52 ± 0.03	0.55 ± 0.05	0.47 ± 0.01	0.49 ± 0.03
Adiponectin (μg/ml)	11	18.1 ± 1.3	17.6 ± 1.0	14.7 ± 1.6	14.8 ± 1.7
	22	18.4 ± 1.5*ab*	18.8 ± 0.9*a*	15.3 ± 0.7*b*	15.7 ± 0.8*b*

Values within a row not sharing the same letter indicate significant difference, p < 0.05.

§ p < 0.05, # p < 0.01, * p < 0.001 indicate significant difference between 11 and 22 weeks.

### Lipid metabolism

**Figure 5:**
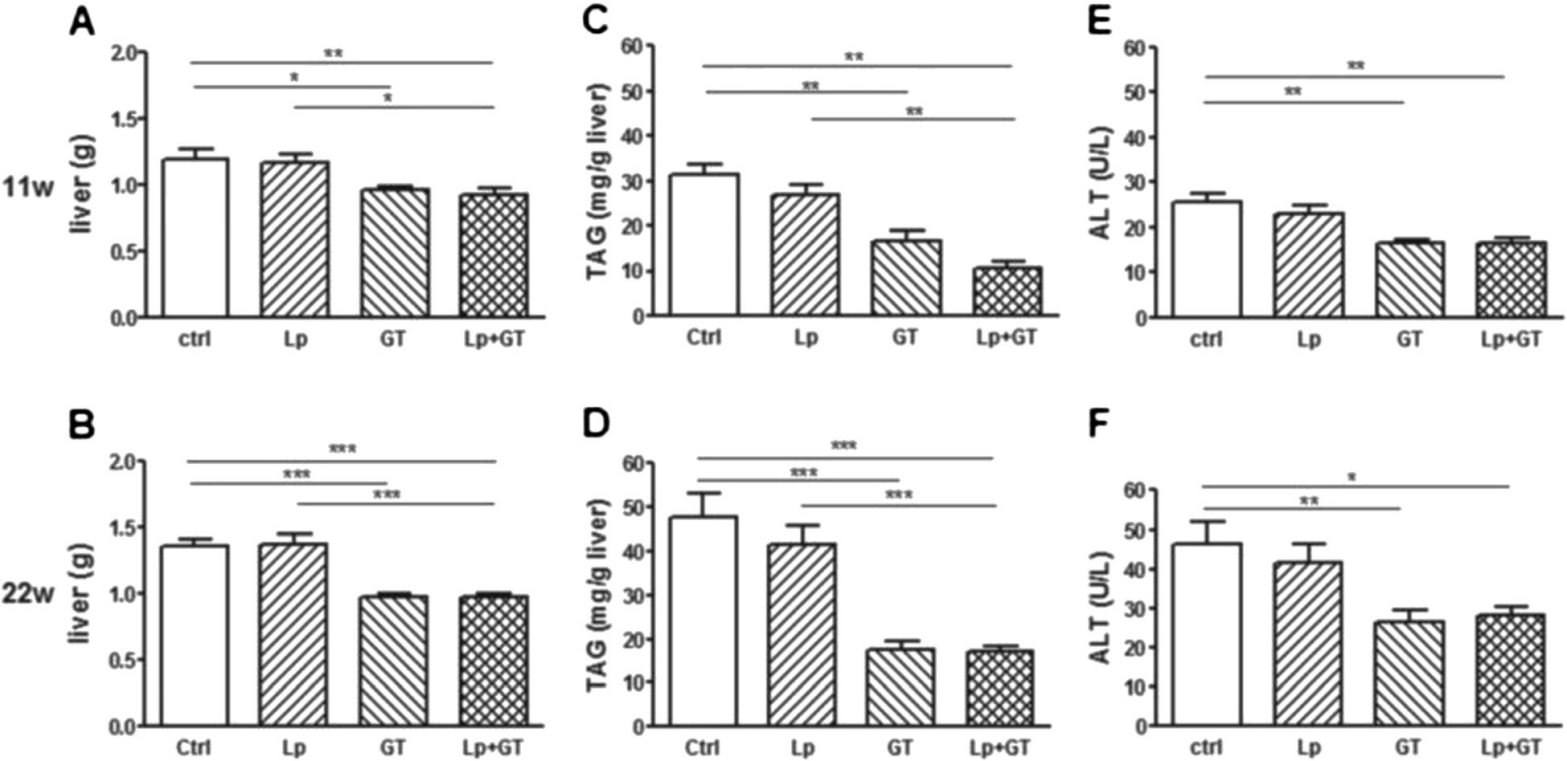
**Improved liver phenotype in mice fed a diet supplemented with green tea.** Liver weights after 11 (**A**) and 22 (**B**) weeks on the different diets. Triacylglycerol (TAG) content in liver after 11 (**C**) and 22 (**D**) weeks, respectively. The concentration of the liver enzyme ALT measured in plasma after 11 (**E**) and 22 (**F**) weeks. Ctrl = high fat control diet (HFD), Lp = HFD + *L. plantarum* in the drinking water, GT = HFD supplemented with 4% green tea powder, Lp + GT = HFD supplemented with 4% green tea powder and *L. plantarum* in the drinking water. Data are means ± SEM for n = 21 at 11 weeks and n = 9–11 at 22 weeks. *p < 0.05, **p < 0.01, ***p < 0.001.

**Figure 6:**
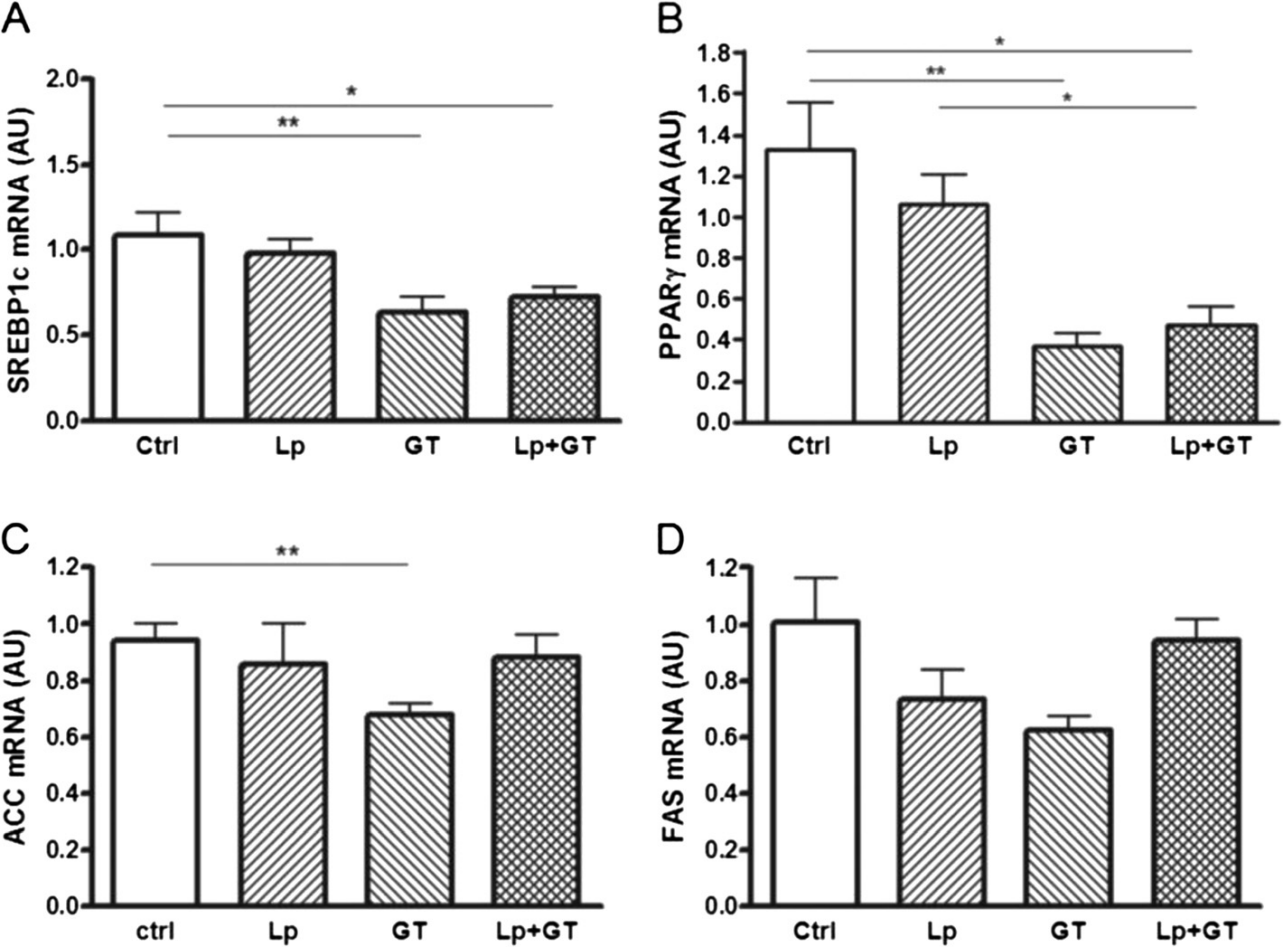
**Decreased lipogenic gene expression in liver of mice fed a diet supplemented with green tea.** Hepatic mRNA expression of sterol regulatory-binding protein 1c (SREBP1c, **A**), peroxisome proliferator-activated receptor γ (PPARγ, **B**), acetyl CoA carboxylase (ACC, **C**) and fatty acid synthase (FAS, **D**) was quantified with real-time PCR after 22 weeks of the different diets. Ctrl = high fat control diet (HFD), Lp = HFD + *L. plantarum* in the drinking water, GT = HFD supplemented with 4% green tea powder, Lp + GT = HFD supplemented with 4% green tea powder and *L. plantarum* in the drinking water. Data are means ± SEM. n = 9–11 at 22 weeks. *p < 0.05, **p < 0.01.

Both the GT and Lp + GT groups had significantly lower plasma TAG compared to control after 22 weeks (Table 2). No differences in plasma TAG were observed at the start of the study. Liver weights were significantly lower in mice from both GT groups compared to control mice after both 11 and 22 weeks (Figure 5A and B). The liver weight was also significantly lower in the Lp + GT group compared to the Lp group. Also the liver TAG content related to liver mass was decreased in the groups fed GT and showed the same picture regarding significance as the liver weight (Figure 5C and D). Between 11 and 22 weeks, there was a general increase in liver TAG accumulation in all groups but the GT group. Moreover, the liver enzyme ALT was decreased in plasma from mice in the GT groups compared to the mice in the control group (Figure 5E and F). However, plasma ALT was generally higher in all groups after 22 weeks compared to 11 weeks. After 22 weeks the mRNA expression of the lipogenic transcription factors SREBP1c and PPARγ was significantly down-regulated in both groups of mice fed GT (Figure 6A and B). The mRNA expression of the lipogenic enzyme ACC was decreased only in mice receiving GT compared to control, and the same trend was observed for the expression of FAS mRNA (Figure 6C and D). The hepatic mRNA expression of PPARα, PGC-1α, CD36, ACADL, LXR, PXR, chREBP, XBP1, PEPCK, CREB and GK is shown in the Additional file 9. A significant difference in the mRNA expression of PPARα, which plays a role in the control of fatty acid oxidation, was observed between mice in the GT group compared to control mice at 22 weeks. Also, a decreased mRNA expression of CD36 and LXR, both involved in the control of lipogenesis, was observed in mice from the Lp + GT group compared to control, after 11 and 22 weeks respectively (Additional file 9). The amount of *Akkermansia* correlated negatively with the TAG content in the liver (rho = −0.44; p = 0.03) and there was also a tendency towards a negative correlation between the total amount of bacteria and liver TAG (rho-0.36; p = 0.07).

### Cholesterol metabolism

**Figure 7:**
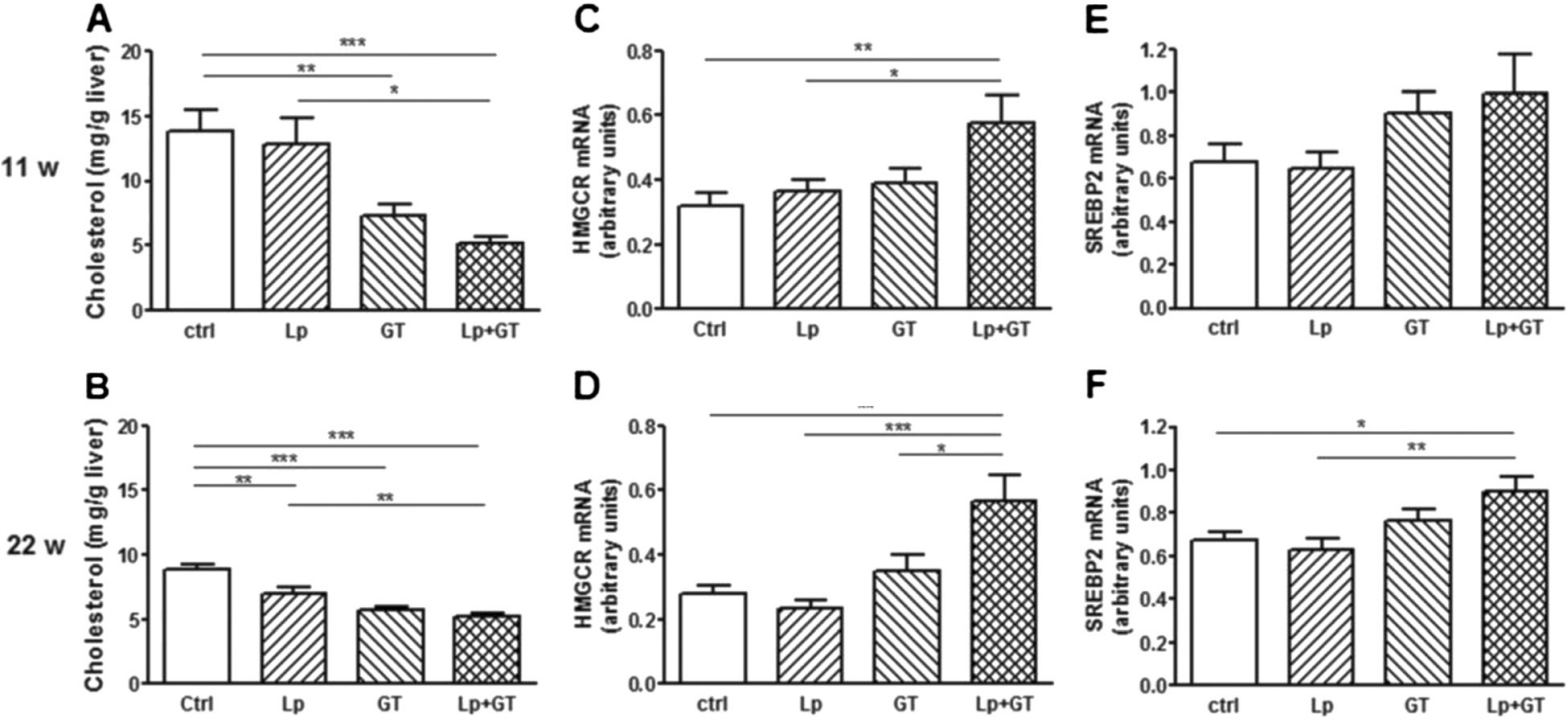
**Altered cholesterol homeostasis in mice fed a diet supplemented with green tea and*Lactobacillus plantarum*.** Total cholesterol content in liver after 11 (**A**) and 22 (**B**) weeks on the different diets. Hepatic mRNA expression of hydroxy-methyl-glutaryl-CoA reductase (HMGCR, **C** and **D**) and sterol regulatory-binding protein 2 (SREBP2, **E** and **F**) after 11 and 22 weeks respectively. Ctrl = high fat control diet (HFD), Lp = HFD + *L. plantarum* in the drinking water, GT = HFD supplemented with 4% green tea powder, Lp + GT = HFD supplemented with 4% green tea powder and *L. plantarum* in the drinking water. Data are means ± SEM. n = 21 at 11 weeks and n = 9–11 at 22 weeks. *p < 0.05, **p < 0.01, ***p < 0.001.

Plasma cholesterol was significantly higher in the Lp + GT group after 11 weeks but after 22 weeks it was significantly lower in both GT groups compared to control (Table 2). No differences in plasma cholesterol were observed between the groups at the start of the study (data not shown). The liver cholesterol content was decreased in both groups receiving GT after 11 weeks, but after 22 weeks it was also decreased in the Lp group compared to control (Figure 7A and B). Liver cholesterol decreased over time in all groups but Lp + GT, which showed unchanged cholesterol content between 11 and 22 weeks. After 11 weeks, the key enzyme in cholesterol synthesis, HMGCoA reductase was upregulated in the Lp + GT group but no significant changes in the mRNA expression of the cholesterol-regulating transcription factor SREBP2 were observed between the groups (Figure 7C and E). However, after 22 weeks, HMGCoA reductase was significantly up-regulated in the Lp + GT group compared to the other groups (Figure 7D) and SREBP2 was upregulated compared to control and Lp (Figure 7F). The hepatic expression of SR-B1, LDLR and CYP7A1, genes involved in reverse cholesterol transport, is shown in Additional file 9. SR-B1, an HDL receptor, was significantly upregulated in mice fed GT compared to control mice. The mRNA expression of the LDL receptor was down-regulated in mice in the GT group compared to control mice but the significance was eliminated with addition of Lp. Total cholesterol excretion was increased in the GT groups compared to control (control: 0.64 ± 0.1, Lp: 0.65 ± 0.11, GT: 1.56 ± 0.11, Lp + GT: 1.55 ± 0.18 mg/mouse/24 h). The hepatic cholesterol content and plasma cholesterol correlated negatively with the total amount of bacteria (rho = −0.43; p = 0.04 and rho = −0.44; p = 0.04 respectively).

### Markers of inflammation

**Figure 8:**
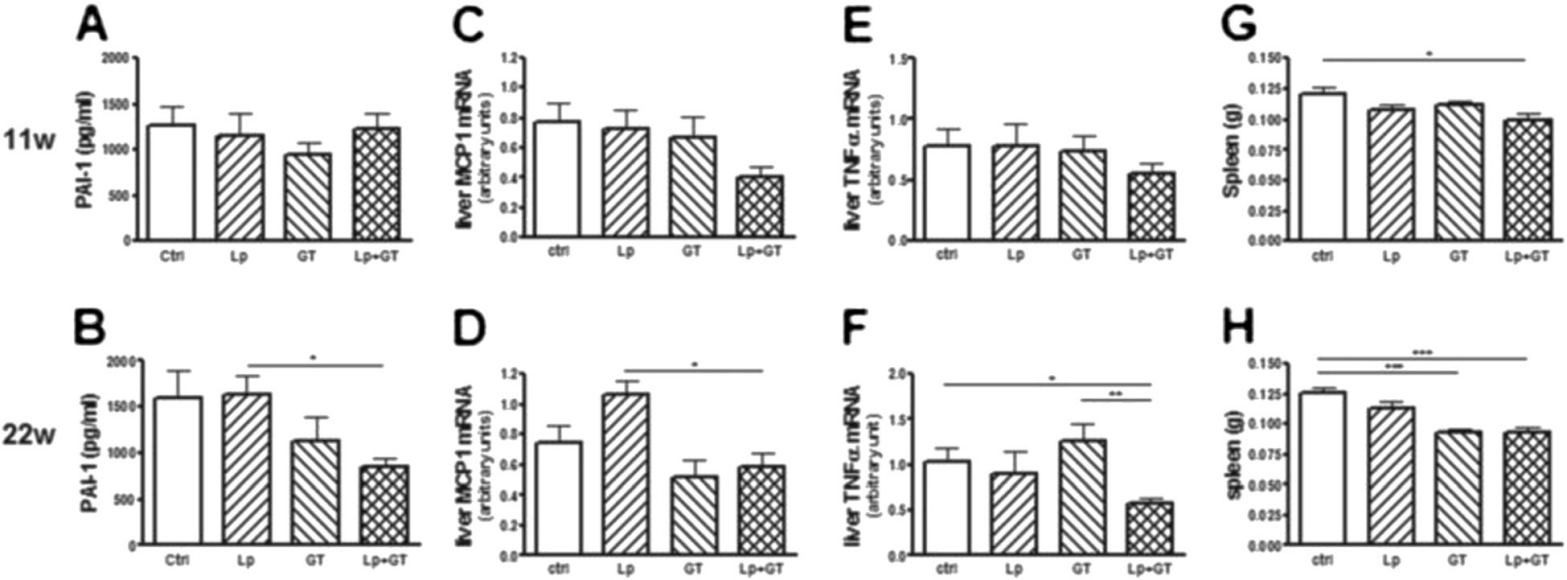
**Decreased inflammatory markers in mice fed a combination of green tea and*Lactobacillus plantarum*.** The inflammatory marker PAI-1 was analyzed in plasma after 11 (**A**) and 22 (**B**) weeks using Luminex Technology. Quantitative real-time PCR was used to analyze the liver mRNA expression of the inflammatory markers monocyte chemoattractant protein 1 (MCP-1, **C** and **D**) and tumor necrosing factor α (TNF-α, **E** and **F**) after 11 and 22 weeks of the study. Spleen masses were analyzed as a reflection of inflammatory activity (**G** and **H**). Ctrl = high fat control diet (HFD), Lp = HFD + *L. plantarum* in the drinking water, GT = HFD supplemented with 4% green tea powder, Lp + GT = HFD supplemented with 4% green tea powder and *L. plantarum* in the drinking water. Data are means ± SEM. n = 21 at 11 weeks and n = 9–11 at 22 weeks. *p < 0.05, **p < 0.01, ***p < 0.001.

Circulating PAI-1 was significantly decreased in mice in the Lp + GT group compared to mice in the Lp group (p < 0.05) after 22 weeks but not after 11 weeks (Figure 8A and B). Plasma PAI-1 was also negatively correlated with *Akkermansia* (rho = −0.49; p = 0.03) at 22 weeks. The cytokines IL-6 and MCP-1 were analyzed in the same multiplex assay as PAI-1, but the plasma concentrations were below the detection limit. The hepatic mRNA expression of MCP-1 and TNF-α showed no differences after 11 weeks but with a tendency of decreased MCP-1 in the Lp + GT group (Figure 8C and E). After 22 weeks MCP-1 decreased in the Lp + GT group compared to the Lp group and TNF-α decreased in the Lp + GT group compared to both the control and the GT group (p < 0.05) (Figure 8D and F). The hepatic mRNA expression of PAI-1, TLR4, MyD88 and F4-80 is shown in Additional file 9. A significant difference in the expression of TLR4 was observed between mice in GT and Lp + GT groups. Moreover, the spleen weights were decreased in the Lp + GT group compared to control group after 11 weeks (p < 0.05) (Figure 8G), and after 22 weeks, mice in the two GT groups had significantly smaller spleens compared to the control group (p < 0.001) (Figure 8H).

### Multivariate data analysis

A PCA of the metabolic data was performed for the four groups of mice. The mice fed green tea clustered separately from the control and Lp groups (data not shown). An x/y plot showed that most of the variability in the model was explained by body fat (data not shown).

**Figure 9:**
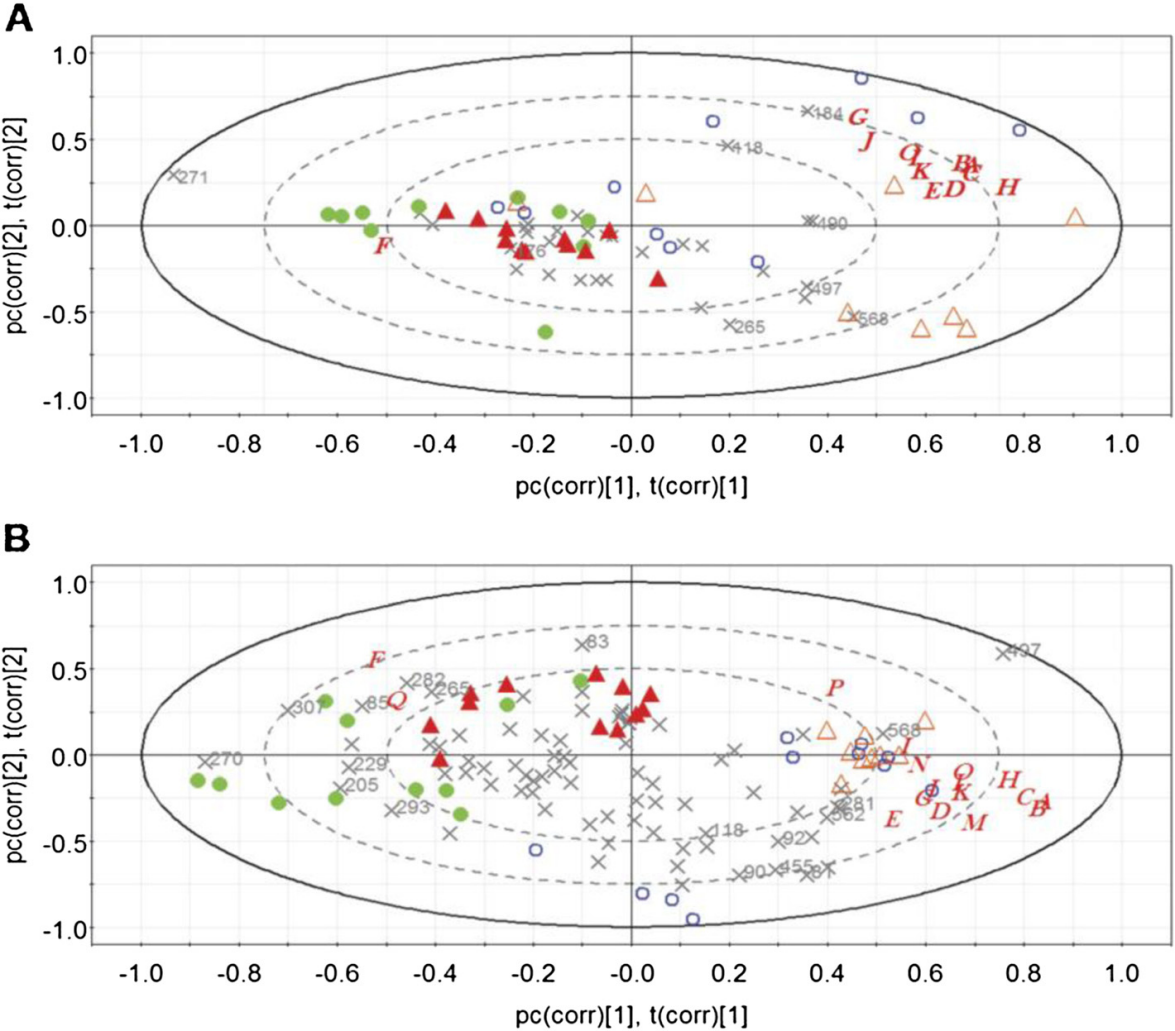
**PLS plot of the intestinal microbiota and analyzed parameters of individual mice at 22 weeks.** Projections to latent structures (PLS) loadings biplot of the microbiota (T-RFs of the T-RFLP profile) and the physiological parameters (A through Q) of the individual mice in the small intestine (**A**) and caecum (**B**). For the small intestine PC1 explained 25.6% and PC2 15.6% of the variation. For caecum the PC1 explained 26.4% and PC2 16.4% of the variation. Blue open circle represents mice in the control group (Ctrl); orange open triangle for the group supplemented with *L. plantarum* DSM 15313 (Lp); green dot for the group supplemented green tea (GT); red triangle for the group supplemented *L. plantarum* DSM 15313 and green tea (Lp + GT). Letters denote metabolic parameters, A, body weight; B, body fat content; C, periovarian white adipose tissue; D, liver weight; E, spleen weight; F, caecum weight; G, liver cholesterol; H, liver TAG; I, plasma ALT; J, plasma cholesterol; K, plasma insulin; L, plasma glucose; M, plasma leptin; N, PPARα mRNA; O, PPARγ mRNA; P, CD36 mRNA; Q, SR-B1 mRNA. Symbol X denotes T-RFs. T-RFs having the biggest influence on the model are marked by numbers indicating the fragment size.

A PCA was applied on the T-RFLP data of the small intestine and the caecum, respectively, combined with the metabolic parameters, in order to reveal differences and similarities between and within the four groups. The combined data for the small intestine showed that mice in the GT and Lp + GT groups displayed more similarity to each other compared to the control and Lp groups (data not shown). The combined caecum data indicate that the mice in the Lp group clustered together while the mice in the three other groups were more different from each other (data not shown). A PLS analysis of the combined data was then performed to reveal any correlation between the T-RFs, i.e. microbiota, and the metabolic parameters. The obtained PLS loadings bi-plots showed that the control and the Lp groups were separated from the GT and Lp + GT groups (Figure 9A and B). Variables located closely together are more likely to have a positive correlation, while variables far away from each other, either along the first or the second principal component, are more likely to be negatively correlated. The T-RFs having the most influence on the model are indicated with their fragment size, and in the small intestine a high abundance of T-RFs 118 and 184 correlated positively to the increase of the metabolic markers while T-RF 271 was negatively correlated (Figure 9A). In the ceacum, T-RFs 270 and 307 correlated negatively with all the metabolic markers except SR-B1 expression and caecum weight while T-RF 568 and 497 correlated positively with the same data (Figure 9B). The T-RF 568 corresponds to the given strain *L. plantarum* DSM 15313 and the positive correlation is probably due to the high abundance of this strain in the Lp group.

## Discussion

The present study shows that green tea powder (GT) alone, or in combination with *L. plantarum* DSM 15313 (Lp), exerts beneficial metabolic effects in C57BL/6J mice fed an HFD. These results are in agreement with and an extension of previous studies of metabolic effects of green tea in rodents. However, in contrast to most previous studies, in which the green tea extract or the phenolic constituent epigallocatechin gallate (EGCG) has been evaluated, we have here studied the effect of whole tea leaves milled to a powder, which is suggested to contain higher quantities of polyphenols compared to a water extract of green tea [45]. Indeed, our analysis of the green tea powder showed a higher amount of catechins compared to a water extract from the same green tea powder. The present study additionally shows that when a tannase active strain of *L. plantarum* was supplemented to the GT-diet (Lp + GT), both the load of lactobacilli and the bacterial diversity increased significantly in the small intestine. It seems likely that the increase in diversity was due to effects exerted by the given *Lactobacillus* strain in combination with GT rather than just the addition of one more species to the microbiota.

The observed reduction in adiposity after green tea intake is in agreement with previous studies, including studies in HFD-fed C57BL/6J mice. Increased faecal lipid excretion is likely to be a major factor contributing to the reduced adiposity. It is known that green tea extracts possess the ability to inhibit intestinal lipases [46]. In addition to lipases, green tea catechins have been shown to inhibit glucose transporters and enzymes involved in carbohydrate digestion, raising the possibility that also carbohydrate absorption could be reduced [47,48]. EGCG has previously been shown to slightly increase energy content of faeces as a consequence of reduced digestibility [14]. There were indications that components of the microbiota might interfere with lipid metabolism. The amount of *Akkermansia* in the small intestine was shown to correlate negatively with the total body fat content, the periovarian fat depots as well as the circulating levels of leptin, suggesting a role for *Akkermansia* in reducing fat accumulation. Interestingly, *Akkermansia muciniphila* was recently shown to be increased in prebiotic-treated *ob/ob* mice which had lower fat mass compared to control *ob/o*b mice [49]. In a study of pregnant women, the number of *Akkermansia* and *Bifidobacterium* was higher in women with normal weight gain compared to those with excessive weight gain [50]. *Akkermansia* has also been shown to be increased in normal weight and post-gastric-bypass individuals compared to obese [51]. In the present study, a negative correlation was also detected between the total number of bacteria and periovarian fat mass. However, it remains to be proven that the correlations point at the cause of the alterations in lipid metabolism.

In accordance with the reduced adiposity after GT intake, GT reduced hepatic lipid content and circulating levels of ALT suggesting that GT either directly, or indirectly, perhaps via an altered microbiota, ameliorates the liver damage imposed by the HFD. The involvement of an altered microbiota is supported by the negative correlation between the amount of *Akkermansia* and the hepatic TAG content. Ma et al. demonstrated that the probiotic mixture of different *Lactobacillus* and *Bifidobacterium* strains (labelled VSL#3) reduced a HFD induced hepatic steatosis in C57BL6 mice [11]. On the other hand Velayudham et al. demonstrated that the same probiotic mixture did not prevent liver steatosis but modulated the progression to liver fibrosis [52]. Here, the supplement with one strain of *L. plantarum* did not have the capacity to reduce the hepatic TAG accumulation. SREBP-1c is a key transcription factor in hepatic lipogenesis and is partly regulated by PPARγ and LXR [53]. The down-regulation of the mRNA expression of SREBP-1c, PPARγ and LXR suggests that decreased hepatic lipogenesis contributes to the reduced hepatic lipid content observed in the mice fed GT. Also, the hepatic fatty acid transporter CD36, another lipogenic target of PPARγ and LXR [53] was shown to be down-regulated in the GT groups compared to control at 11 weeks, however, the decrease compared to control was abolished after 22 weeks. In addition, the hepatic mRNA expression of the lipogenic enzymes ACC and FAS was reduced and trended towards a diminuition, respectively, in mice fed GT alone compared to control mice, indicating a decreased de novo lipogenesis in these mice. Surprisingly, the decreased expression of the lipogenic enzymes could not be detected in mice from the Lp + GT group. The expression of the transcription factor PPARα was down-regulated in mice in the GT group compared to control mice, indicating a decreased hepatic fatty acid oxidation as well. The mechanisms behind this finding need further elucidation. It should be emphasized that this is a large screening of the alterations induced by the dietary supplements and more detailed analyses, including expression at the protein level, need to be undertaken.

The choice of the present strain of probiotics is partly based upon its ability to increase the barrier effect of the gut-mucosa [54] but mainly on its ability to degrade polyphenols as tannins, and produce compounds as substituted phenyl propionic acids, phenyl valeric acids and benzoic acid derivates [26]. These compounds are more easily absorbed than longer molecular chains of phenolics, and are often also more bioactive [55,56]. They can have anti-inflammatory effects as well as antimicrobial effects. One hypothesis is that Lp in the large intestine is able to convert polyphenols in GT to more easily absorbed metabolites with antioxidative effects in organs such as the liver. Generally, polyphenols possess powerful antimicrobial activities [27] which can have growth suppressing effects on many bacteria but which are better endured by others.

Feeding mice with Lp alone did not affect inflammatory markers. A reduced spleen weight was observed already after 11 weeks in the Lp + GT group and Lp + GT significantly reduced the inflammatory tone at 22 weeks, both systemically, as indicated by reduced circulating PAI-1 and decreased spleen weight [57], as well as locally in the liver, as shown by decreased mRNA expression of TNF-α and MCP-1. This, together with the higher number of *Lactobacillus* both in caecum and small intestine in the Lp + GT group support the hypothesis of *Lactobacillus* converting polyphenols to more active anti-inflammatory components. The higher number of lactobacilli in the Lp group and higher diversity in the GT group in the ceacum seemed not to affect the inflammatory markers at 11 weeks. The effect seen after 22 weeks might partly be explained by an increased number of bacterial taxa over time in all groups since the number of T-RFs increased at 22 weeks while no difference in diversity between the groups was seen in ceacum.

It has previously been shown that a probiotic-supplemented diet decreases levels or expression of liver TNF-α in animal models. Ma et al. [11] found a decreased expression of TNF-α in the liver of HFD-fed C57BL/6 mice when the diet was supplemented with the probiotic VSL#3 mixture. Furthermore, in an acute liver injury model in rats, TNF-α levels decreased in the liver when rats had been pre-treated with *L. plantarum* DSM 15313 before inducing liver injury [58]. Also, green tea extract alone has been shown to reduce hepatic mRNA levels of both TNF-α and MCP-1 as well as NFκB binding activity [59]. Additionally, a negative correlation between *Akkermanisia* and plasma PAI-1 was seen, indicating that *Akkermansia* may have a beneficial influence on the inflammatory state. It has been shown in germ-free mice inoculated with *Akkermansia municiphila* Muc^T^ that the colonization altered the mucosal gene expression towards a profile involved in immune responses and cell fate, which led to the conclusion that the tested strain of *Akkermansia* modulated pathways involved in establishing homeostasis for basal metabolism and immune tolerance towards commensal bacteria [60].

Dietary administration of GT reduced plasma cholesterol as well as hepatic cholesterol content. The supplementation with Lp alone had a significant cholesterol-lowering effect in the liver after 22 weeks and additionally the total number of bacteria in the small intestine was negatively correlated with both liver and plasma cholesterol. An increased faecal excretion of cholesterol was observed in the GT groups, indicating that this is at least one of the mechanisms underlying the cholesterol-lowering effect of GT administration. EGCG as well as other polyphenols have previously been shown to inhibit cholesterol absorption in rodents [61,62]. The underlying mechanisms are not fully elucidated but green tea catechins have been suggested to reduce the absorption of cholesterol from the intestine by reducing the solubility of cholesterol in mixed micelles [61]. SREBP2, the major regulator in cholesterol biosynthesis, and its downstream target HMG-CoA reductase, were up-regulated when GT was combined with Lp. An explanation for the up-regulated cholesterol biosynthesis might be a response to the very efficient GT-induced cholesterol excretion in an attempt of the system to restore cholesterol homeostasis. This rescue mechanism of cholesterol is further supported by the upregulation of the hepatic HDL receptor SR-B1 mRNA in the GT groups.

The lower fasting plasma glucose and insulin, resulting in a lower HOMA index, as well as the lower levels of fructosamine, mirroring the blood glucose concentration over several weeks, indicate a more insulin sensitive state in the mice fed GT. In contrast, the oral glucose tolerance test showed deteriorated glucose elimination despite increased insulin secretion in the GT groups, implying decreased oral glucose tolerance compared to the control group and the Lp group. The cause for this discrepancy is not known.

A component of the microbiota that seems to be of relevance for several of the metabolic effects studied here is *Akkermansia*. *Akkermansia muciniphila* is a newly described species which has been shown to be an efficient mucin degrader found in the intestines of humans and animals [63] and it has been associated with healthy gut mucosa [64,65]. However, the amount of *Akkermansia* in the small intestine did not differ between the groups indicating that it is not the diet *per se* but instead the response of the specific microbiota in an individual that might be of importance. It is clear from the present results that the dietary supplements GT and Lp exercise effects on the composition of the microbiota in both the small intestine and in caecum as well as on metabolism. However, a key-question that remains to be answered is whether the changes in gut-microbiota affect the metabolic markers or whether the changes in the gut-microbiota result from metabolic alterations. With the assumption that certain components of the microbiota exert metabolic effects, it is clear from the present results that the microbiota of the individual mice varied widely in spite of the fact that they are from an inbred strain. Especially the individuals of the control group differ while the dietary supplements appear to standardize the microbiota to some degree. Surprisingly, the standardization differed between the small intestine and caecum, i.e. green tea made the microbiota of the individual mice more uniform in the former while Lp did the same in the latter. The multivariate analysis revealed that certain, relatively few components of the microbiota (T-RFs) in both the small intestine and in caecum, had a considerably higher effect on the correlation model built for comparing T-RFLP-data with metabolic test-parameters, some showing positive correlations and some negative. It was also clear that GT and Lp in some cases affected the abundance of these bacterial components differently. One of these critical components (T-RF) could be identified as *Akkermansia.* However, the fact that several correlations have been found between *Akkermansia* and metabolic parameters do not necessarily implicate a causal role for this taxum but at least reflects that markers for inflammation and lipid metabolism are linked to the microbiota, and especially the microbiota of the small intestine. Mice in the Lp + GT group had higher bacterial diversity in the small intestine compared to both control and the GT group. The higher diversity may as such be a positive health factor or at least a health marker. It was shown in humans that the bacterial diversity of the gut microbiota was higher in lean individuals than in obese ones [66] and that neonates with low diversity at one week of age had increased risk for developing atopic eczema at 18 months of age [67]. As expected the number of T-RFs were smaller in the small intestine compared to caecum as the sampling were done close to the pylorus. In humans, the diversity in the jejunum has been shown to be lower than in the colon [68]. The T-RFLP method was chosen here even if it has a lower resolution compared to high through-put sequencing, but it gives comparable results to pyrotag sequencing regarding diversity measures [69].

## Conclusions

An extensive battery of parameters has been analyzed in the present study in order to evaluate the effects of two different dietary supplements alone and in combination on gut microbiota and metabolism. It seems clear that some of the metabolic effects described in this study were accounted for by GT alone, whereas others, notably the reduced inflammation and up-regulation of genes regulating cholesterol synthesis, were only significantly observed with the combination of Lp and GT. We propose that GT acts as a potent source for dietary polyphenols, promoting the growth of a healthy, anti-inflammatory intestinal microbiota and that addition of a tannase active strain of *L. plantarum* results in certain synbiotic effects. In addition, certain bacterial fractions of the microbiota correlate positively or negatively with risk factors for type 2 diabetes. It is of interest to identify the unknown fractions and to clarify if *Akkermansia* or some of these unknown correlating fractions are the actual cause of the observed metabolic alterations.

## Abbreviations

ALT: Alanine aminotransferase; AUC: Area under curve; cfu: Colony forming units; EGCG: Epigallocatechin gallate; GT: Green tea powder; HFD: High-fat diet; HOMA: Homeostasis model assessment; IL-6: Interleukin 6; Lp: Lactobacillus plantarum; MCP-1: Monocyte chemoattractant protein 1; NEFA: Non esterified fatty acids; PAI-1: Plasminogen activator inhibitor 1; PCA: Principal component analysis; PLS: Partial Least Squares Projections to Latent Structures; qPCR: Quantitative PCR; RAPD: Random amplified polymorphic DNA; T-RF: Terminal restriction fragment; T-RFLP: Terminal restriction fragment length polymorphism; TAG: Triacylglycerol.

## Competing interests

The authors declare that they have no competing interests.

## Authors' contributions

UA participated in planning and coordinating the study, conducted in vivo experiments, collected data, performed data analysis, interpreted data and drafted the manuscript. CO participated in the planning of the study, microbial analysis, interpretation of data and in writing the manuscript. JX performed microbial analysis, the statistical correlations analysis and PCA analysis of the microbiota and participated in the manuscript preparation. CF participated in the planning of the study, data collection and critical review of the manuscript. SL collected data and performed data analysis. KS participated in the planning of the study, data collection and critical review of the manuscript. SA participated in the planning of the study. CH participated in the planning of the study, interpretation of the data and critical review of the manuscript. GM conceived the hypothesis, planned the study, interpreted data and participated in the writing of the manuscript. KB conceived the hypothesis, planned and coordinated the study, collected data, interpreted data and drafted the manuscript. All authors read and approved the final version of the manuscript.

## Supplementary Material

Additional file 1 Nutritional composition of green tea powder. Click here for file

Additional file 2 Composition of the experimental diets. Click here for file

Additional file 3 Additional information about the PCR-based methods. Click here for file

Additional file 4 **Primer sequences used in the qPCR for quantification of*Lactobacillus*, total bacteria,*Enterobacteriaceae*and*Akkermansia*in the small intestinal tissue samples.** Click here for file

Additional file 5 Primer sequences used in the qPCR analyses of the liver tissue. Click here for file

Additional file 6 Viable count of lactobacilli in caecum of the different groups. Click here for file

Additional file 7 Bacterial diversity in caecum after 11 and 22 weeks. Click here for file

Additional file 8 Oral glucose tolerance test at week 21. Click here for file

Additional file 9 Hepatic mRNA expression. Click here for file

## References

[B1] Shaw JE Sicree RA Zimmet PZ Global estimates of the prevalence of diabetes for 2010 and 2030 Diabetes Res Clin Pract 2010 87 4 14 10.1016/j.diabres.2009.10.007 19896746

[B2] Smith BW Adams LA Nonalcoholic fatty liver disease and diabetes mellitus, pathogenesis and treatment Nat Rev Endocrinol 2011 7 456 465 10.1038/nrendo.2011.72 21556019

[B3] Backhed F Ding H Wang T Hooper LV Koh GY Nagy A Semenkovich CF Gordon JI The gut microbiota as an environmental factor that regulates fat storage Proc Natl Acad Sci USA 2004 101 15718 15723 10.1073/pnas.0407076101 15505215 PMC524219

[B4] Larsen N Vogensen FK van den Berg FW Nielsen DS Andreasen AS Pedersen BK Al-Soud WA Sorensen SJ Hansen LH Jakobsen M Gut microbiota in human adults with type 2 diabetes differs from non-diabetic adults PLoS One 2010 5 e9085 10.1371/journal.pone.0009085 20140211 PMC2816710

[B5] Turnbaugh PJ Backhed F Fulton L Gordon JI Diet-induced obesity is linked to marked but reversible alterations in the mouse distal gut microbiome Cell Host Microbe 2008 3 213 223 10.1016/j.chom.2008.02.015 18407065 PMC3687783

[B6] Cani PD Delzenne NM Gut microflora as a target for energy and metabolic homeostasis Curr Opin Clin Nutr Metab Care 2007 10 729 734 10.1097/MCO.0b013e3282efdebb 18089955

[B7] Cani PD Neyrinck AM Fava F Knauf C Burcelin RG Tuohy KM Gibson GR Delzenne NM Selective increases of bifidobacteria in gut microflora improve high-fat-diet-induced diabetes in mice through a mechanism associated with endotoxaemia Diabetologia 2007 50 2374 2383 10.1007/s00125-007-0791-0 17823788

[B8] Membrez M Blancher F Jaquet M Bibiloni R Cani PD Burcelin RG Corthesy I Mace K Chou CJ Gut microbiota modulation with norfloxacin and ampicillin enhances glucose tolerance in mice FASEB J 2008 22 2416 2426 10.1096/fj.07-102723 18326786

[B9] Cani PD Bibiloni R Knauf C Waget A Neyrinck AM Delzenne NM Burcelin R Changes in gut microbiota control metabolic endotoxemia-induced inflammation in high-fat diet-induced obesity and diabetes in mice Diabetes 2008 57 1470 1481 10.2337/db07-1403 18305141

[B10] Esposito E Iacono A Bianco G Autore G Cuzzocrea S Vajro P Canani RB Calignano A Raso GM Meli R Probiotics reduce the inflammatory response induced by a high-fat diet in the liver of young rats J Nutr 2009 139 905 911 10.3945/jn.108.101808 19321579

[B11] Ma X Hua J Li Z Probiotics improve high fat diet-induced hepatic steatosis and insulin resistance by increasing hepatic NKT cells J Hepatol 2008 49 821 830 10.1016/j.jhep.2008.05.025 18674841 PMC2588670

[B12] Rubino F R’Bibo SL del Genio F Mazumdar M McGraw TE Metabolic surgery, the role of the gastrointestinal tract in diabetes mellitus Nat Rev Endocrinol 2010 6 102 109 10.1038/nrendo.2009.268 20098450 PMC2999518

[B13] Cabrera C Artacho R Gimenez R Beneficial effects of green tea–a review J Am Coll Nutr 2006 25 79 99 16582024 10.1080/07315724.2006.10719518

[B14] Klaus S Pultz S Thone-Reineke C Wolfram S Epigallocatechin gallate attenuates diet-induced obesity in mice by decreasing energy absorption and increasing fat oxidation Int J Obes (Lond) 2005 29 615 623 10.1038/sj.ijo.0802926 15738931

[B15] Bose M Lambert JD Ju J Reuhl KR Shapses SA Yang CS The major green tea polyphenol, (−)-epigallocatechin-3-gallate, inhibits obesity, metabolic syndrome, and fatty liver disease in high-fat-fed mice J Nutr 2008 138 1677 1683 18716169 10.1093/jn/138.9.1677 PMC2586893

[B16] Ikeda I Hamamoto R Uzu K Imaizumi K Nagao K Yanagita T Suzuki Y Kobayashi M Kakuda T Dietary gallate esters of tea catechins reduce deposition of visceral fat, hepatic triacylglycerol, and activities of hepatic enzymes related to fatty acid synthesis in rats Biosci Biotechnol Biochem 2005 69 1049 1053 10.1271/bbb.69.1049 15914933

[B17] Murase T Nagasawa A Suzuki J Hase T Tokimitsu I Beneficial effects of tea catechins on diet-induced obesity, stimulation of lipid catabolism in the liver Int J Obes Relat Metab Disord 2002 26 1459 1464 10.1038/sj.ijo.0802141 12439647

[B18] Wolfram S Raederstorff D Wang Y Teixeira SR Elste V Weber P TEAVIGO (epigallocatechin gallate) supplementation prevents obesity in rodents by reducing adipose tissue mass Ann Nutr Metab 2005 49 54 63 10.1159/000084178 15735368

[B19] Wolfram S Wang Y Thielecke F Anti-obesity effects of green tea, from bedside to bench Mol Nutr Food Res 2006 50 176 187 10.1002/mnfr.200500102 16470636

[B20] Nagao T Hase T Tokimitsu I A green tea extract high in catechins reduces body fat and cardiovascular risks in humans Obesity (Silver Spring) 2007 15 1473 1483 10.1038/oby.2007.176 17557985

[B21] Dulloo AG Duret C Rohrer D Girardier L Mensi N Fathi M Chantre P Vandermander J Efficacy of a green tea extract rich in catechin polyphenols and caffeine in increasing 24-h energy expenditure and fat oxidation in humans Am J Clin Nutr 1999 70 1040 1045 10584049 10.1093/ajcn/70.6.1040

[B22] Nagao T Meguro S Hase T Otsuka K Komikado M Tokimitsu I Yamamoto T Yamamoto K A catechin-rich beverage improves obesity and blood glucose control in patients with type 2 diabetes Obesity (Silver Spring) 2009 17 310 317 19008868 10.1038/oby.2008.505

[B23] Koo SI Noh SK Green tea as inhibitor of the intestinal absorption of lipids, potential mechanism for its lipid-lowering effect J Nutr Biochem 2007 18 179 183 10.1016/j.jnutbio.2006.12.005 17296491 PMC1852441

[B24] Barthelmebs L Divies C Cavin JF Knockout of the p-coumarate decarboxylase gene from Lactobacillus plantarum reveals the existence of two other inducible enzymatic activities involved in phenolic acid metabolism Appl Environ Microbiol 2000 66 3368 3375 10.1128/AEM.66.8.3368-3375.2000 10919793 PMC92157

[B25] Osawa R Kuroiso K Goto S Shimizu A Isolation of tannin-degrading lactobacilli from humans and fermented foods Appl Environ Microbiol 2000 66 3093 3097 10.1128/AEM.66.7.3093-3097.2000 10877812 PMC92117

[B26] Vaquero I Marcobal A Munoz R Tannase activity by lactic acid bacteria isolated from grape must and wine Int J Food Microbiol 2004 96 199 204 10.1016/j.ijfoodmicro.2004.04.004 15364474

[B27] Aziz NH Farag SE Mousa LA Abo-Zaid MA Comparative antibacterial and antifungal effects of some phenolic compounds Microbios 1998 93 43 54 9670554

[B28] Lee JH Shim JS Lee JS Kim JK Yang IS Chung MS Kim KH Inhibition of pathogenic bacterial adhesion by acidic polysaccharide from green tea (Camellia sinensis) J Agric Food Chem 2006 54 8717 8723 10.1021/jf061603i 17090112

[B29] Ankolekar C Johnson D Pinto MD Johnson K Labbe R Shetty K Inhibitory potential of Tea polyphenolics and influence of extraction time against helicobacter pylori and lack of inhibition of beneficial lactic acid bacteria J Med Food 2011 14 1321 1329 10.1089/jmf.2010.0237 21663484

[B30] Ahn Y-J Sakanaka S Kim M-J Kawamura T Fujisawa T Mitsuoka T Effect of green tea extract on growth of intestinal bacteria Microbial Ecol Health Dis 1990 3 335 338 10.3109/08910609009140256

[B31] Karlsson CL Molin G Fak F Johansson Hagslatt ML Jakesevic M Hakansson A Jeppsson B Westrom B Ahrne S Effects on weight gain and gut microbiota in rats given bacterial supplements and a high-energy-dense diet from fetal life through to 6 months of age Br J Nutr 2011 106 887 895 10.1017/S0007114511001036 21450114

[B32] Gustafsson RJ Ohlsson B Benoni C Jeppsson B Olsson C Mucosa-associated bacteria in two middle-aged women diagnosed with collagenous colitis World J Gastroenterol 2012 18 1628 1634 10.3748/wjg.v18.i14.1628 22529692 PMC3325529

[B33] Falk A Olsson C Ahrne S Molin G Adawi D Jeppsson B Ileal pelvic pouch microbiota from two former ulcerative colitis patients, analysed by DNA-based methods, were unstable over time and showed the presence of Clostridium perfringens Scand J Gastroenterol 2007 42 973 985 10.1080/00365520701204238 17613928

[B34] Zheng D Alm EW Stahl DA Raskin L Characterization of universal small-subunit rRNA hybridization probes for quantitative molecular microbial ecology studies Appl Environ Microbiol 1996 62 4504 4513 8953722 10.1128/aem.62.12.4504-4513.1996 PMC168277

[B35] Hall TA BioEdit, a user-friendly biological sequence alignment editor and analysis program for windows 95/98/NT Nucleic Acids Symp 1999 41 95 98

[B36] Cole JR Wang Q Cardenas E Fish J Chai B Farris RJ Kulam-Syed-Mohideen AS McGarrell DM Marsh T Garrity GM Tiedje JM The ribosomal database project, improved alignments and new tools for rRNA analysis Nucleic Acids Res 2009 37 D141 D145 10.1093/nar/gkn879 19004872 PMC2686447

[B37] Krebs CJ Ecological methodology 1998 2 California: Benjamin Cummings

[B38] Magurran AE Ecological diversity and its measurements 1996 London: Champman and Hall

[B39] Vandesompele J De Preter K Pattyn F Poppe B Van Roy N De Paepe A Speleman F Accurate normalization of real-time quantitative RT-PCR data by geometric averaging of multiple internal control genes Genome Biol 2002 3 RESEARCH0034 12184808 10.1186/gb-2002-3-7-research0034 PMC126239

[B40] Matthews DR Hosker JP Rudenski AS Naylor BA Treacher DF Turner RC Homeostasis model assessment, insulin resistance and beta-cell function from fasting plasma glucose and insulin concentrations in man Diabetologia 1985 28 412 419 10.1007/BF00280883 3899825

[B41] Best DJ RD Algorithm AS 89, the upper tail probabilities of spearmans rho Applied Statistics 1975 24 377 379 10.2307/2347111

[B42] Myles Hollander MWD Nonparametric statistical methods 1973 New York: John Wiley & Sons

[B43] Hothorn THK Van de Wiel MA Zeileis A A lego system for conditional interference Am Stat 2006 24 257 263

[B44] Hothorn THK Van de Wiel MA Zeileis A Implementing a class of permutation tests, The coin package J Stat Softw 2008 28 1 23

[B45] Friedman M Levin CE Choi SH Kozukue E Kozukue N HPLC analysis of catechins, theaflavins, and alkaloids in commercial teas and green tea dietary supplements, Comparison of water and 80% ethanol/water extracts J Food Sci 2006 71 C328 C337 10.1111/j.1750-3841.2006.00090.x

[B46] Juhel C Armand M Pafumi Y Rosier C Vandermander J Lairon D Green tea extract (AR25) inhibits lipolysis of triglycerides in gastric and duodenal medium in vitro J Nutr Biochem 2000 11 45 51 10.1016/S0955-2863(99)00070-4 15539342

[B47] Kobayashi Y Suzuki M Satsu H Arai S Hara Y Suzuki K Miyamoto Y Shimizu M Green tea polyphenols inhibit the sodium-dependent glucose transporter of intestinal epithelial cells by a competitive mechanism J Agric Food Chem 2000 48 5618 5623 10.1021/jf0006832 11087528

[B48] Naz S Siddiqi R Dew TP Williamson G Epigallocatechin-3-gallate inhibits lactase but is alleviated by salivary proline-rich proteins J Agric Food Chem 2011 59 2734 2738 10.1021/jf103072z 21348516

[B49] Everard A Lazarevic V Derrien M Girard M Muccioli GG Neyrinck AM Possemiers S Van Holle A Francois P de Vos WM Responses of gut microbiota and glucose and lipid metabolism to prebiotics in genetic obese and diet-induced leptin-resistant mice Diabetes 2011 60 2775 2786 10.2337/db11-0227 21933985 PMC3198091

[B50] Santacruz A Collado MC Garcia-Valdes L Segura MT Martin-Lagos JA Anjos T Marti-Romero M Lopez RM Florido J Campoy C Sanz Y Gut microbiota composition is associated with body weight, weight gain and biochemical parameters in pregnant women Br J Nutr 2010 104 83 92 10.1017/S0007114510000176 20205964

[B51] Zhang H DiBaise JK Zuccolo A Kudrna D Braidotti M Yu Y Parameswaran P Crowell MD Wing R Rittmann BE Krajmalnik-Brown R Human gut microbiota in obesity and after gastric bypass Proc Natl Acad Sci USA 2009 106 2365 2370 10.1073/pnas.0812600106 19164560 PMC2629490

[B52] Velayudham A Dolganiuc A Ellis M Petrasek J Kodys K Mandrekar P Szabo G VSL#3 probiotic treatment attenuates fibrosis without changes in steatohepatitis in a diet-induced nonalcoholic steatohepatitis model in mice Hepatology 2009 49 989 997 10.1002/hep.22711 19115316 PMC3756672

[B53] Zhou J Febbraio M Wada T Zhai Y Kuruba R He J Lee JH Khadem S Ren S Li S Hepatic fatty acid transporter Cd36 is a common target of LXR, PXR, and PPARgamma in promoting steatosis Gastroenterology 2008 134 556 567 10.1053/j.gastro.2007.11.037 18242221

[B54] Osman N Adawi D Ahrne S Jeppsson B Molin G Probiotics and blueberry attenuate the severity of dextran sulfate sodium (DSS)-induced colitis Dig Dis Sci 2008 53 2464 2473 10.1007/s10620-007-0174-x 18274903

[B55] Mulder TP Rietveld AG van Amelsvoort JM Consumption of both black tea and green tea results in an increase in the excretion of hippuric acid into urine Am J Clin Nutr 2005 81 256S 260S 15640488 10.1093/ajcn/81.1.256S

[B56] Roowi S Stalmach A Mullen W Lean ME Edwards CA Crozier A Green tea flavan-3-ols, colonic degradation and urinary excretion of catabolites by humans J Agric Food Chem 2010 58 1296 1304 10.1021/jf9032975 20041649

[B57] Siegmund B Rieder F Albrich S Wolf K Bidlingmaier C Firestein GS Boyle D Lehr HA Loher F Hartmann G Adenosine kinase inhibitor GP515 improves experimental colitis in mice J Pharmacol Exp Ther 2001 296 99 105 11123368

[B58] Osman N Adawi D Ahrne S Jeppsson B Molin G Endotoxin- and D-galactosamine-induced liver injury improved by the administration of lactobacillus. Bifidobacterium and blueberry Dig Liver Dis 2007 39 849 856 10.1016/j.dld.2007.06.001 17652039

[B59] Park HJ Lee JY Chung MY Park YK Bower AM Koo SI Giardina C Bruno RS Green tea extract suppresses NFkappaB activation and inflammatory responses in diet-induced obese rats with nonalcoholic steatohepatitis J Nutr 2012 142 57 63 10.3945/jn.111.148544 22157544

[B60] Derrien M Van Baarlen P Hooiveld G Norin E Muller M de Vos WM Modulation of mucosal immune response, tolerance, and proliferation in mice colonized by the mucin-degrader akkermansia muciniphila Front Microbiol 2011 2 166 21904534 10.3389/fmicb.2011.00166 PMC3153965

[B61] Raederstorff DG Schlachter MF Elste V Weber P Effect of EGCG on lipid absorption and plasma lipid levels in rats J Nutr Biochem 2003 14 326 332 10.1016/S0955-2863(03)00054-8 12873714

[B62] Sobolova L Skottova N Vecera R Urbanek K Effect of silymarin and its polyphenolic fraction on cholesterol absorption in rats Pharmacol Res 2006 53 104 112 10.1016/j.phrs.2005.09.004 16275123

[B63] Derrien M van Passel MW van de Bovenkamp JH Schipper RG de Vos WM Dekker J Mucin-bacterial interactions in the human oral cavity and digestive tract Gut Microbes 2010 1 254 268 10.4161/gmic.1.4.12778 21327032 PMC3023607

[B64] Swidsinski A Dorffel Y Loening-Baucke V Theissig F Ruckert JC Ismail M Rau WA Gaschler D Weizenegger M Kuhn S Acute appendicitis is characterised by local invasion with Fusobacterium nucleatum/necrophorum Gut 2011 60 34 40 10.1136/gut.2009.191320 19926616

[B65] Png CW Linden SK Gilshenan KS Zoetendal EG McSweeney CS Sly LI McGuckin MA Florin TH Mucolytic bacteria with increased prevalence in IBD mucosa augment in vitro utilization of mucin by other bacteria Am J Gastroenterol 2010 105 2420 2428 10.1038/ajg.2010.281 20648002

[B66] Turnbaugh PJ Hamady M Yatsunenko T Cantarel BL Duncan A Ley RE Sogin ML Jones WJ Roe BA Affourtit JP A core gut microbiome in obese and lean twins Nature 2009 457 480 484 10.1038/nature07540 19043404 PMC2677729

[B67] Wang M Karlsson C Olsson C Adlerberth I Wold AE Strachan DP Martricardi PM Aberg N Perkin MR Tripodi S Reduced diversity in the early fecal microbiota of infants with atopic eczema J Allergy Clin Immunol 2008 121 129 134 10.1016/j.jaci.2007.09.011 18028995

[B68] Wang M Ahrne S Jeppsson B Molin G Comparison of bacterial diversity along the human intestinal tract by direct cloning and sequencing of 16S rRNA genes FEMS Microbiol Ecol 2005 54 219 231 10.1016/j.femsec.2005.03.012 16332321

[B69] Pilloni G Granitsiotis MS Engel M Lueders T Testing the limits of 454 pyrotag sequencing, reproducibility, quantitative assessment and comparison to T-RFLP fingerprinting of aquifer microbes PLoS One 2012 7 e40467 10.1371/journal.pone.0040467 22808168 PMC3395703

