# Supplementary material for: Green tea powder and Lactobacillus plantarum affect gut microbiota, lipid metabolism and inflammation in high-fat fed C57BL/6J mice
Source: Nutr Metab (Lond). 2012 Nov 26;9:105. doi: 10.1186/1743-7075-9-105 (PMC3538623; doi:10.1186/1743-7075-9-105)
Supplement: Additional file 1 — Nutritional composition of green tea powder. [file 1743-7075-9-105-S1.docx]

**Additional file 1**

Nutritional composition of green tea powder (Premium Powdered Sencha)

| Protein, g/100g | 29.2 |
| --- | --- |
| Fat, g/100g | 4.2 |
| Carbohydrates, g/100g | 30.1 |
| Dietary fiber, g/100g | 27.9 |
| water soluble fiber, % of dietary fiber | 17.0 |
| non-water soluble fiber, % of dietary fiber | 83.0 |
| Ashes, g/100g | 5.1 |
| Water, g/100g | 3.5 |
| Energy content, kJ/100g | 1163.5 |

Polyphenol composition of green tea powder (Premium Powdered Sencha)

| **Substance** | Water extraction  mg/g green tea powder | | Methanol:water extraction (60:40)  mg/g green tea powder | |
| --- | --- | --- | --- | --- |
|  | **Mean** | **SD** | **Mean** | **SD** |
| **Flavan-3-ol** |  |  |  |  |
| Catechin | 1.50 | 0.20 | 1.64 | 0.24 |
| Epicatechin | 2.86 | 0.23 | 0.92 | 0.04 |
| Epicatechin-3-gallate | 5.73 | 0.10 | 10.34 | 0.43 |
| Gallocatechin | 4.86 | 0.22 | 4.52 | 1.33 |
| Epigallocatechin | 10.45 | 0.49 | 12.81 | 0.50 |
| Epigallocatechingallate (EGCG) | 14.20 | 0.94 | 20.74 | 1.39 |
| **Total** | **39.60** |  | **50.97** |  |
| **Phenolic acids** |  |  |  |  |
| Galloyl quinic acid | 0.72 | 0.06 | 0.52 | 0.14 |
| 3-caffeoyl-quinic acid | 0.08 | 0.01 | 0.08 | 0.00 |
| 5-caffeoyl-quinic acid | 0.02 | 0.00 | 0.02 | 0.01 |
| 4-p-coumarylquinic acid | 1.77 | 0.11 | 1.51 | 0.08 |
| **Total** | **2.59** |  | **2.13** |  |
| **Purin alkaloids** |  |  |  |  |
| Caffeine | 21.03 | 0.93 | 27.89 | 1.24 |
| Theobromine | 0.24 | 0.00 | 0.22 | 0.01 |
| **Total** | **21.27** |  | **28.11** |  |
| **Flavonols** |  |  |  |  |
| Quercetin-galactosylrutinoside | 1.14 | 0.04 | 1.07 | 0.16 |
| Quercetin-glucosylrutinoside | 1.51 | 0.02 | 1.39 | 0.08 |
| Myricetin 3-O-rhamnosylglucoside | 0.16 | 0.01 | 0.15 | 0.01 |
| Myricetin 3-O-galactoside | 0.46 | 0.06 | 0.64 | 0.05 |
| Myricetin 3-O-glucoside | 0.80 | 0.05 | 1.05 | 0.11 |
| Quercetin 3-O-dirhamnosylglucoside | 0.80 | 0.07 | 0.62 | 0.06 |
| Kaempferol-3-o-galactosylrutinoside | 0.98 | 0.05 | 0.91 | 0.02 |
| Kaempferol-3-o-rutinoside | 0.20 | 0.01 | 0.17 | 0.06 |
| Apigenin 6.8- C-dipentoside | 0.02 | 0.00 | 0.02 | 0.01 |
| Quercetin 3-O-acylglucoside | 0.07 | 0.01 | 0.09 | 0.02 |
| **Total** | **6.14** |  | **6.11** |  |
| **Dimerer/trimerer(proanthocyanidiner)** |  |  |  |  |
| Procyanidindimer | 2.21 | 0.14 | 2.53 | 0.27 |
| Gallocatechin catechingallate | 0.30 | 0.02 | 0.53 | 0.04 |
| Procyanidintrimer | 0.14 | 0.05 | 0.17 | 0.02 |
| Digallocatechin-catechin | 0.00 | 0.00 | 0.04 | 0.01 |
| **Total** | **2.65** |  | **3.27** |  |

**Extraction, separation and analysis of polyphenols in green tea powder**

Analysis of phenolic components and alkaloids in the green tea powder was performed by HPLC according to the methods of Lin et al. (Lin LZ, Chen P, Harnly JM: **New phenolic components and chromatographic profiles of green and fermented teas.** J Agric Food Chem 2008, 56:8130-8140), with some modifications. Briefly, for each triplicate, 100 mg sample was extracted in 5 ml of either methanol:water (60:40, v/v) or pure water. The extraction was performed using an Ultrasonic sonicator Bandelin Sonorex DT100H (Berlin, Germany) for 60 min at 30°C. The extracts were centrifuged at 5000g for 5 min at 4°C and filtered through a 17 mm PVDF syringe filter, 0.45 µm (VWR, Sweden), and thereafter 10 µl of the extract was injected onto the chromatographic column Luna C18(2) 3.0 µm 100 x 3mm equipped with a security guard Ultra (Phenomenex, Inc. USA). The eluent consisted of solvent A (0.1% formic acid in water) and solvent B (0.1% formic acid in 50% methanol, 50% acetonitrile, v/v), with a binary gradient 5% B (0–5 min), 5–26% B (5–25 min), 26–65% B (25–35 min), 65% B (35-37 min), 65-5% B (37-38 min) and 5% B (38-45 min). Flow rate 0.425 ml/min. The analyses were made on an Agilent 1200 HPLC-DAD-ESI/MS (Agilent Technologies, Waldbronn, Germany). Evaluation of data were performed with LC/MSD Chemstation Rev. B.04.03(16).
