# Supplementary material for: Green tea powder and Lactobacillus plantarum affect gut microbiota, lipid metabolism and inflammation in high-fat fed C57BL/6J mice
Source: Nutr Metab (Lond). 2012 Nov 26;9:105. doi: 10.1186/1743-7075-9-105 (PMC3538623; doi:10.1186/1743-7075-9-105)
Supplement: Additional file 2 — Composition of the experimental diets. [file 1743-7075-9-105-S2.docx]

**Additional file 2**

Composition of the experimental diets

|  | Ctrl | Lp | GT | Lp+GT |
| --- | --- | --- | --- | --- |
| Ingredients | g/kg | | | |
| Casein 80 mesh | 233 | 233 | 220,6 | 220,6 |
| L-cystein | 3.5 | 3.5 | 3.5 | 3.5 |
| Corn starch | 84.8 | 84.8 | 72.5 | 72.5 |
| Maltodextrin | 116.5 | 116.5 | 116.1 | 116.1 |
| Sucrose | 201.4 | 201.4 | 200.7 | 200.7 |
| Cellulose | 58.3 | 58.3 | 46.9 | 46.9 |
| Soybean oil | 29.1 | 29.1 | 29.0 | 29.0 |
| Lard | 206.9 | 206.9 | 204.5 | 204.5 |
| Mineral mix | 11.7 | 11.7 | 11.6 | 11.6 |
| DiCalciumphosphate | 15.1 | 15.1 | 15.1 | 15.1 |
| Calcium carbonate | 6.4 | 6.4 | 6.4 | 6.4 |
| Potassium citrate | 19.2 | 19.2 | 19.2 | 19.2 |
| Vitamin mix | 11.7 | 11.7 | 11.6 | 11.6 |
| Choline bitartrate | 2.3 | 2.3 | 2.3 | 2.3 |
| Green tea powder | - | - | 40 | 40 |
| Calculated energy content, kJ/g | 19.8 | 19.8 | 19.7 | 19.7 |
|  | Energy % | | | |
| Protein | 20 | | | |
| Carbohydrates | 35 | | | |
| Fat | 45 | | | |
| Total fiber % | 5.8 | | | |
