# Supplementary material for: Green tea powder and Lactobacillus plantarum affect gut microbiota, lipid metabolism and inflammation in high-fat fed C57BL/6J mice
Source: Nutr Metab (Lond). 2012 Nov 26;9:105. doi: 10.1186/1743-7075-9-105 (PMC3538623; doi:10.1186/1743-7075-9-105)
Supplement: Additional file 3 — Additional information about the PCR-based methods. [file 1743-7075-9-105-S3.docx]

**Additional file 3**

**RAPD**

Identification by Random amplification of polymorphic DNA (RAPD) was performed as described earlier (Quednau et al. 1998). Isolated pure cultures putatively identified as *Lactobacillus* spp. were regrown on Rogosa agar. Growth was collected and washed twice with sterile water. After washing 250 µl of water and 8-10 glass beads were added to the tube and shaken on an Eppendorf Mixer 5432 (Eppendorf, Hamburg, Germany) to disintegrate the cells. Two µl of the preparation was used in RAPD-PCR using a 9-mer primer (5`-acgcgccct-3´). The PCR parameters were initially 4 cycles with 94°C for 45 s, 30°C for 2 min and 72°C for 30 s. This was followed by 26 cycles with 95°C for 5 s, 36°C for 30 s and 72° for 30 s (with 1 s extension per cycle) and the PCR ended with an elongation step at 72°C for 10 min. The PCR-products were analyzed by agarose gel electrophoresis and isolates showing the same band pattern as the PCR-product from *L. plantarum* DSM 15313 were putatively identified as *L. plantarum* DSM 15313.

**Amplification and T-RFLP analysis**

For T-RFLP analysis the universal bacterial 16S RNA genes were amplified by ENV1 (5´ - agagtttgatiitggctcag - 3´) and ENV2 primers (5´- cggitaccttgttacgactt - 3´). The ENV1 primer was fluorescently labelled with FAM at the 5´-end. The reaction mixture contained 5 µl of 10X polymerase chain reaction (PCR) buffer (100 mM Tris-HCl, 15 mM MgCl_2_, 500 mM KCl, pH 8.3), 200 mM of each deoxyribonucleotide phosphate, 2.5 U of Taq DNA polymerase (Roche Diagnostics, GmbH, Mannheim, Germany), 0.4 µM of the labelled forward primer and 0.2 µM of ENV2 primer. Amplification was performed with an Eppendorf Mastercycler (Eppendorf) using one denaturation step at 94°C for 3 min. followed by 30 cycles with 1 min at 94°C, 45 seconds at 50°C, 2 min. at 72°C. Reaction ended with an elongation step at 72°C for 7 min and then cooled down to 4°C. Correct sizes amplicons were verified by agarose gel electrophoresis. Triplicate reactions were prepared for each sample and the amplified products were pooled and purified with the MinElute PCR Purification Kit (Qiagen). The amount of DNA was measured by Nanodrop ND-1000 Spectrophotometer (Nanodrop Technologies, Wilmington, USA) and 200 ng of DNA was digested with 15 U of restriction endonuclease *Msp*I (Fermentas Life Science, Burlington, Canada) for 4-5 hours in a total volume of 15 or 20 µl. For caecum samples *Alu*I (Fermentas Life Science) was also used. Samples were analysed on an ABI 3130xl (Applied Biosystems, Foster City, CA, USA) and to each sample the GeneScan™- 600LIZ^®^ standard (20-600 bp) was added (Applied Biosystems). The Local Southern method was chosen for size calling.

**PCR and cloning of the 16S rRNA genes**

For cloning of the small intestinal samples the bacterial 16S rRNA genes were amplified by unlabelled primer ENV1 and ENV2 as described above for T-RFLP analysis except that that the amount of primers was 0.2 µM of each and that the PCR was run for 25 cycles. Six reactions were run from each sample and then pooled. The pooled PCR products were verified by agarose gel electrophoresis and then purified using the Wizard^®^ SV Gel and PCR Clean-Up System (Promega, Madison, USA). The DNA was eluted in 20 µl of ddH_2_O (distilled, autoclaved and UV-light treated water) according to manufacturer’s instruction. The purified PCR products were ligated into pGEM-T vector system II (Promega, Madison, USA) and then transformed into *E. coli* JM109 high efficiency competent cells according to manufacturer´s instructions. The cells were cultivated on Luria-Bertani (LB) agar plates supplemented with ampicillin (100 µg/ml; Sigma, St. Louis, USA), IPTG (0.5 mM) and X-gal (100 µg/ml). Colonies were blue/white screened and white colonies were randomly selected from the plates and recultivated on LB-agar with ampicillin. Growth was collected and stored in glycerol containing freezing buffer and at -80°C.

**SYBR Green qPCR assay**

Quantitative PCR was run in a Mastercycler® ep realplex 1.5 real-time PCR system (Eppendorf) separately for *Lactobacillus*, *Akkermansia*, *Enterobacteriaceae* and total bacteria. The qPCR reaction mixture consisted of 10 µl of 2X Platinum®SYBR® Green qPCR SuperMix-UDG (Invitrogen A/S, Taastrup, Denmark), 0.5 µM each of the forward and the reverse primer (see additional file 3), 2 µl of template DNA and ddH_2_O to 20 µl. Triplicate of standards, negative controls and duplicate of samples were prepared in a sterile 96-well Polypropylene microplate (Eppendorf). The qPCR was run under the following conditions. Initially the temperature were set to 50°C for 2 min, followed by 95°C for 2 min. 40 cycles were then run with the following parameters: 95°C for 15 s, primer annealing for 30 s and elongation at 72°C for 30 s. Finally a melting curve analysis was performed with denaturation at 95°C for 15s and 60°C for 15s followed by a temperature gradient from 60-95°C for 20 min and a final denaturation at 95°C for 15s. For amplification of the total bacteria the elongation time was set for 45s at 72°C.

**Standard preparation for qPCR of bacteria**

The genomic DNA was extracted from pure cell cultures of *Lactobacillus plantarum* CCUG 35035 and *Escherichia coli* CCUG 29300 (Culture collection, University of Gothenburg, Sweden). The former was used as template for lactobacilli and total bacteria and the latter for *Enterobacteriaceae*. *L. plantarum* was grown in MRS broth (Merck KGaA, Darmstadt, Germany) and *E. coli* in Brain heart infusion (BHI) (OXOID, Hampshire, England). *L. plantarum* was incubated anaerobically at 37°C for 2 days and *E. coli* aerobically for 24 hours. Cells were then spun down and the DNA was extracted by using QIAamp® DNA Stool Kit (Qiagen) according to the manufacturer’s protocol and finally the DNA was eluted in 100 µl AE buffer (Qiagen). For the preparation of a standard for *Akkermansia muciniphila* a clone obtained from the caecum content of a mouse was used. Previously the bacterial 16S rRNA genes had been cloned from the caecum content from a C57BL6 mouse. The 16s rRNA genes had been amplified by the primers ENV1 (5´-AGAGTTTGATIITGGCTCAG-3´) and ENV 2 (5´-CGGITACCTTGTTACGACTT-3´) and cloned into pGEM vector system II as described above. The clone had then been single strand sequenced for the first  430 bp of the 16S rRNA gene at MWG (Ebersberg, Germany). The clone had then been identified by using the Ribosomal Database and the option “sequence match” and the sequence was 100 % similar to the *Akkermansia muciniphila* (AY271254A). The clone was grown in LB broth supplemented with ampicillin. The bacterial DNA was extracted by using QIAprep® Miniprep kit (Qiagen). The target DNA fragments of lactobacilli, *Enterobacteriaceae*, *Akkermansia* and total bacteria were amplified by using primer sets listed in additional file 3. Six reactions were run for 25 cycles for each primer set. The PCR reaction mixture contained 2.5 µl of 10 x PCR reaction buffer (500 mM Tris-HCl, 100 mM KCl, 50 mM (NH_4_)_2_SO_4_, 20 mM MgCl_2_, pH 8.3), 0.2 mM of each deoxyribonucleotide triphosphate, 0.2 µM each primer, 2.5 U of FastStart Taq DNA polymerase (Roche Diagnostics, Mannheim, Germany) and 2 µl of template DNA in a total volume of 25 µl. The parameters for the PCR were denaturation at 95ºC for 4 min followed by 25 cycles with 95ºC for 15s, annealing for 30s at 58-61ºC s and elongation at 72ºC for 30s (additional file 3). The correct sizes of the PCR products were verified by agarose gel electrophoresis. Purification of the PCR products and the cloning was performed as described above. The four different clones were then recultivated in LB-ampicillin broth at 37°C overnight. The plasmid DNA was extracted by using QIAprep® Miniprep kit (Qiagen). The concentration of the plasmid DNA was measured by Nanodrop ND-1000 (Nanodrop Technologies, Wilmington, USA). The copy number of each plasmid was calculated. For preparation of the standards a ten-fold dilution series was made of the extracted plasmid DNA in TE buffer (10 mM Tris, 1mM EDTA, pH 8.0) supplemented with 0.1 ug/µl Herring sperm DNA (VWR International, West Chester, PA, USA).

**Reference**

Quednau M, Ahrne S, Petersson AC, Molin G: **Identification of clinically important species of Enterococcus within 1 day with randomly amplified polymorphic DNA (RAPD).** Curr Microbiol 1998, **36:**332-336.
