# Supplementary material for: Green tea powder and Lactobacillus plantarum affect gut microbiota, lipid metabolism and inflammation in high-fat fed C57BL/6J mice
Source: Nutr Metab (Lond). 2012 Nov 26;9:105. doi: 10.1186/1743-7075-9-105 (PMC3538623; doi:10.1186/1743-7075-9-105)
Supplement: Additional file 4 — Primer sequences used in the qPCR for quantification ofLactobacillus, total bacteria,EnterobacteriaceaeandAkkermansiain the small intestinal tissue samples. [file 1743-7075-9-105-S4.docx]

**Additional file 4**

Primer sequences used in the qPCR for quantification of *Lactobacillus*, total bacteria, *Enterobacteriaceae* and *Akkermansia* in the small intestinal tissue samples.

| Name of primer | Sequence (5'―3') | Target group | size (bp)* | Annealing temp. (°C) | Reference |
| --- | --- | --- | --- | --- | --- |
| Lact-F | AGCAGTAGGGAATCTTCCA | *Lactobacillus* | 341 | 61 | Walter et al. 2001 |
| Lact-R | CACCGCTACACATGGAG |  |  |  | Heilig et al. 2002 |
| Uni331-F | TCCTACGGGAGGCAGCAGT | Total bacteria | 466 | 58 | Nadkarni et al. 2002 |
| Uni797-R | GGACTACCAGGGTATCTAATCCTGTT |  |  |  |  |
| Eco1457-F | CATTGACGTTACCCGCAGAAGAAGC | *Entero-bacteriaceae* | 195 | 60 | Bartosch et al. 2004 |
| Eco1652-R | CTCTACGAGACTCAAGCTTGC |  |  |  |  |
| AM1-F | CAGCACGTGAAGGTGGGGAC | *Akkermansia* | 327 | 60 | Collado et al. 2007 |
| AM2-R | CCTTGCGGTTGGCTTCAGAT |  |  |  |  |

* Size of amplicons in base pairs

**References**

Bartosch S, Fite A, Macfarlane GT, McMurdo ME: [**Characterization of bacterial communities in feces from healthy elderly volunteers and hospitalized elderly patients by using real-time PCR and effects of antibiotic treatment on the fecal microbiota**.](http://www.ncbi.nlm.nih.gov/pubmed/15184159) *Appl Environ Microbiol*. 2004, **70:**3575-81.

Collado MC, Derrien M, Isolauri E, de Vos WM, Salminen S: **Intestinal integrity and Akkermansia muciniphila, a mucin-degrading member of the intestinal microbiota present in infants, adults, and the elderly**. *Appl Environ Microbiol*. 2007, **73:**7767-70. Epub 2007 Oct 12.

Heilig HG, Zoetendal EG, Vaughan EE, Marteau P, Akkermans AD, de Vos WM:

[**Molecular diversity of Lactobacillus spp. and other lactic acid bacteria in the human intestine as determined by specific amplification of 16S ribosomal DNA.**](http://www.ncbi.nlm.nih.gov/pubmed/11772617) *Appl Environ Microbiol*. 2002, **68:**114-23.

Nadkarni MA, Martin FE, Jacques NA, Hunter N: [**Determination of bacterial load by real-time PCR using a broad-range (universal) probe and primers set**.](http://www.ncbi.nlm.nih.gov/pubmed/11782518) Microbiology. 2002, **148:**257-66.

Walter J, Hertel C, Tannock GW, Lis CM, Munro K, Hammes WP: [**Detection of Lactobacillus, Pediococcus, Leuconostoc, and Weissella species in human feces by using group-specific PCR primers and denaturing gradient gel electrophoresis.**](http://www.ncbi.nlm.nih.gov/pubmed/11375166) *Appl Environ Microbiol*. 2001, **6**7:2578-85.
