# Supplementary material for: Green tea powder and Lactobacillus plantarum affect gut microbiota, lipid metabolism and inflammation in high-fat fed C57BL/6J mice
Source: Nutr Metab (Lond). 2012 Nov 26;9:105. doi: 10.1186/1743-7075-9-105 (PMC3538623; doi:10.1186/1743-7075-9-105)
Supplement: Additional file 5 — Primer sequences used in the qPCR analyses of the liver tissue. [file 1743-7075-9-105-S5.docx]

**Additional file 5**

Primer sequences used in the qPCR analyses of the liver tissue.

| **TaqMan**  **(Applied Biosystems)** | **Assays-on-demand ID** |  |
| --- | --- | --- |
| TNFα | Mm00443258_m1 |  |
| IL-6 | Mm00446190_m1 |  |
| MCP-1 | Mm99999056_m1 |  |
| PAI-1 | Mm01204469_m1 |  |
| CD36 | Mm01135198_m1 |  |
| LXRα | Mm00443454_m1 |  |
| PXR | Mm00803095_m1 |  |
| CREB | Mm00501607_m1 |  |
| PGC1α | Mm00447183_m1 |  |
| XBP-1 | Mm00457359_m1 |  |
| ACADL | Mm00599660_m1 |  |
| chREBP | Mm00498811_m1 |  |
| F4-80 | Mm00802530_m1 |  |
| Cyp7a1 | Mm00484152_m1 |  |
|  |  |  |
|  |  |  |
| **SYBR green chemistry** | **Forward 5’-3’** | **Reverse 5’-3'** |
| SREBP1c | GGA GCC ATG GAT TGC | GGA AGT CAC TGT CTT |
| SREBP2 | TGT GCG CTC TCG TTT | GTA TAG AAG ACG GCC |
| HMGCR | GCA GTC AGT GGG AAC | CGG CTT CAC AAA CCA |
| ACCα | AGA ATC TGG CTG CAT | TGG TAG ACT GCC CGT |
| FAS | TGG TGA ATT GTC TCC | CAC GTT CAT CAC GAG |
| PPARγ2 | CTG TTT TAT GCT GTT ATG GGT GAA | GCA CCA TGC TCT GGG TCA A |
| PPARα | GGT CTT AAC CGG CCC | AAA CGC AAC GTA GAG |
| LDLR | GCC GAC CTG ATG AAT | GCA GTG ATG TTC ACG |
| PEPCK | TAT CTG GAG GAC CAG | TAG ATC TCA GCG CAT |
| GK | TTG GAC CTT TTT CAC | CCA AGA CAG GCC TTC |
| TLR4 | GCC TTT CAG GGA ATT AAG CTC C | AGA TCA ACC GAT GGA CGT GTA A |
| MyD88 | AGA ACA GAC AGA CTA TCG GCT | CGC CGA CAC CTT TTC TCA AT |
| GAPDH | TGC CCC CAT GTT TGT | TTG CTG ACA ATC TTG |
| RPS29 | GGA GTC ACC CAC GGA AGT | TCC ATT CAA GGT CGC TTA GTC |

**Abbreviations:**

MCP1, monocyte chemoattractant protein 1; TNFα, tumor necrosing factor α; PAI-1, plasminogen activator inhibitor-1; CD36, cluster of differentiation 36; LXR α, liver x receptor α; PXR, pregnane x receptor; CREB, cAMP response element binding protein; PGC1α, peroxisome proliferator-activated receptor γ coactivator 1-α; XBP1, X-box binding protein 1; ACADL, acyl-CoA dehydrogenase long-chain; ChREBP carbohydrate-responsive element binding protein, CYP7A1, cholesterol 7α-hydroxylase; GAPDH, glyceraldehyde-3-phosphate dehydrogenase; RPS29, ribosomal protein S29; PPARγ2, peroxisome proliferator-activated receptor γ2; HMGCR, hydroxy-methyl-glutaryl-CoA reductase; SREBP, sterol regulatory-binding protein; ACC, acetyl CoA carboxylase; FAS, fatty acid synthase; LDLR, low density lipoprotein receptor; PEPCK, phosphoenolpyruvate carboxykinase, GK, glucokinase; TLR4, toll-like receptor 4; MTD88, myeloid differentiation primary response gene
