# Supplementary material for: Green tea powder and Lactobacillus plantarum affect gut microbiota, lipid metabolism and inflammation in high-fat fed C57BL/6J mice
Source: Nutr Metab (Lond). 2012 Nov 26;9:105. doi: 10.1186/1743-7075-9-105 (PMC3538623; doi:10.1186/1743-7075-9-105)
Supplement: Additional file 6 — Viable count of lactobacilli in caecum of the different groups. [file 1743-7075-9-105-S6.docx]

**Additional file 6**

Viable count of lactobacilli in caecum of the different groups.

Data are presented as log cfu/g and values are shown as medians and 25^th^ and 75^th^ percentiles. Ctrl=high fat control diet (HFD), Lp=HFD+*L. plantarum* in the drinking water, GT=HFD supplemented with 4% green tea powder, Lp+GT=HFD supplemented with 4% green tea powder and *L. plantarum* in the drinking water.

|  | Ctrl | Lp | GT | Lp+GT |
| --- | --- | --- | --- | --- |
| 11 weeks | 7.32 (7.17 – 7.74) (n=8) | 8.52 (7.74 – 8.87) ^**^ (n=9) | 7.93 (7.58 – 8.27) (n=10) | 8.13 (7.81 – 8.21) (n=9) |
| 22 weeks | 8.24 (7.57 – 8.53) (n=11) | 8.45 (8.35 – 8.65) (n=9) | 8.29 (7.82 – 8.71) a (n=11) | 8.77 (8.57 – 8.87)^**^ (n=12) |

**) Denotes significant difference (p<0.01) compared to the control at the same time point. a) denotes significant difference compared to Lp+GT (p=0.05).
