# Supplementary material for: Green tea powder and Lactobacillus plantarum affect gut microbiota, lipid metabolism and inflammation in high-fat fed C57BL/6J mice
Source: Nutr Metab (Lond). 2012 Nov 26;9:105. doi: 10.1186/1743-7075-9-105 (PMC3538623; doi:10.1186/1743-7075-9-105)
Supplement: Additional file 7 — Bacterial diversity in caecum after 11 and 22 weeks. [file 1743-7075-9-105-S7.docx]

**Additional file 7**

**Bacterial diversity in caecum after 11 and 22 weeks**

Diversity values given as Shannon and Simpson indices for T-RFLP data, and are based on the relative peak area of the total area when using restriction endonucleases *Msp*1 and *Alu*1. Ctrl=high fat control diet (HFD), Lp=HFD+*L. plantarum* in the drinking water, GT=HFD supplemented with 4% green tea powder, Lp+GT=HFD supplemented with 4% green tea powder and *L. plantarum* in the drinking water.

| Time point of sampling | Group | Shannon index | | Simpson index | |
| --- | --- | --- | --- | --- | --- |
|  |  | *Msp*1 | *Alu*1 | *Msp*1 | *Alu*1 |
| 11 weeks | Ctrl (n=5) | 1,99 (1,95 - 2,12) | 1,78 (1,72 - 1,92) | 4,73 (4,17 - 4,97) | 3,81 (3,63 - 4,51) |
|  | Lp (n=10) | 2,21 (2,14 - 2,85) | 2,20 (2,05 - 2,85) | 4,78 (4,29 - 12,74) | 4,79 (4,31 - 13,32) |
|  | GT (n=9) | 2,53 (2,31 - 2,81)** | 2,49 (2,34 - 2,71)*** | 6,26 (5,57 - 9,48) | 6,67 (5,49 - 10,14)** |
|  | Lp+GT (n=5) | 2,25 (2,21 - 2,30) | 2,30 (2,20 - 2,35) | 4,39 (4,23 - 4,91) | 4,72 (4,43 - 5,29) |
| 22 weeks | Ctrl (n=11) | 2,19 (2,03 - 2,84) | 2,33 (2,12 -2,67) | 4,37 (4,17 - 12,01) | 5,78 (4,88 - 9,97) |
|  | Lp (n=9) | 2,07 (1,98 - 2,18) | 2,19 (2,13 - 2,28) | 5,11 (4,82 - 5,57) | 5,57 (5,19 - 6,38) |
|  | GT (n=11) | 2,41 (2,16 - 2,69) | 2,54 (2,36 -2,88) | 7,48 (5,69 - 9,83) | 9,01 (6,60 - 12,04) |
|  | Lp+GT (n=12) | 2,30 (2,26 - 2,44) | 2,42 (2,27 - 2,50) | 6,41 (5,32 - 7,65) | 8,08 (6,71 -8,78) |

Values are shown as median and 25^th^ and 75^th^ percentiles.* denotes significant difference compared to control (p < 0.05); ** denotes significant difference compared to control (p < 0.01); *** denotes significant difference compared to control (p < 0.001)
