# Supplementary material for: Green tea powder and Lactobacillus plantarum affect gut microbiota, lipid metabolism and inflammation in high-fat fed C57BL/6J mice
Source: Nutr Metab (Lond). 2012 Nov 26;9:105. doi: 10.1186/1743-7075-9-105 (PMC3538623; doi:10.1186/1743-7075-9-105)
Supplement: Additional file 8 — Oral glucose tolerance test at week 21. [file 1743-7075-9-105-S8.docx]

**Additional file 8**


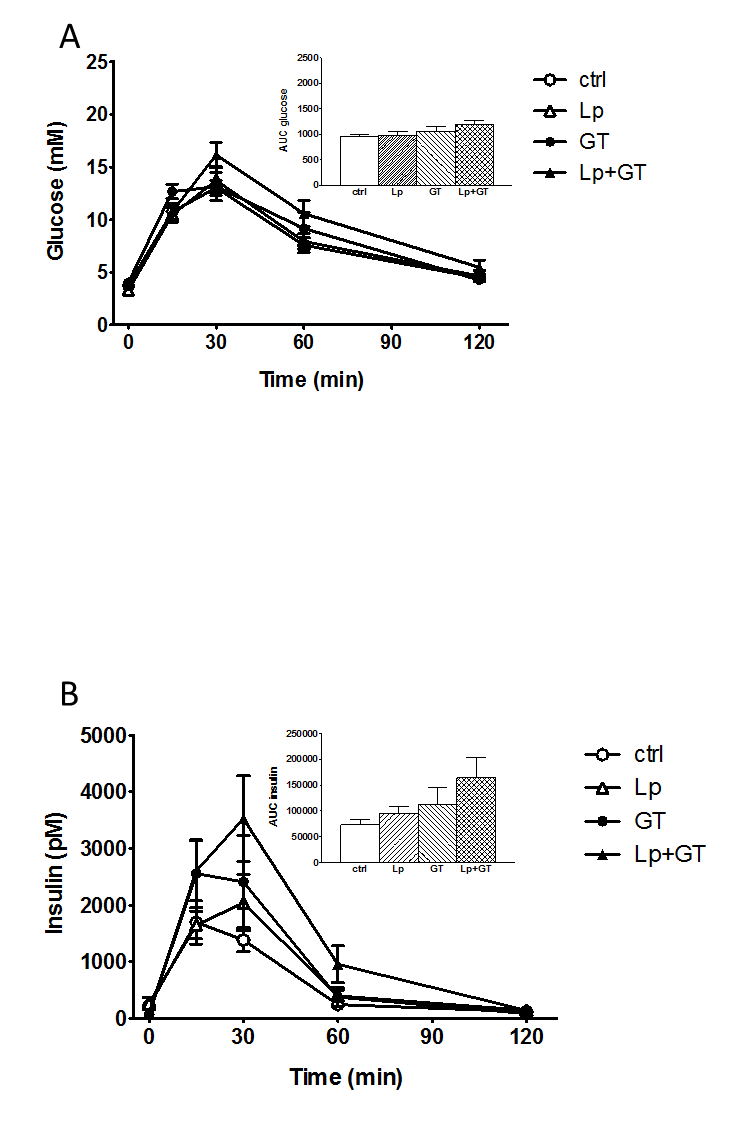


**Oral glucose tolerance test at week 21**

**(**A) Plasma glucose and (B) insulin concentrations in an oral glucose tolerance test performed after 21 weeks of the different diets. Ctrl=high fat control diet (HFD), Lp=HFD+*L. plantarum* in the drinking water, GT=HFD supplemented with 4% green tea powder, Lp+GT=HFD supplemented with 4% green tea powder and *L. plantarum* in the drinking water. Data are means ± SEM for 9-12 mice/group.
