# Supplementary material for: Green tea powder and Lactobacillus plantarum affect gut microbiota, lipid metabolism and inflammation in high-fat fed C57BL/6J mice
Source: Nutr Metab (Lond). 2012 Nov 26;9:105. doi: 10.1186/1743-7075-9-105 (PMC3538623; doi:10.1186/1743-7075-9-105)
Supplement: Additional file 9 — Hepatic mRNA expression. [file 1743-7075-9-105-S9.docx]

**Additional file 9**

Hepatic mRNA expression (arbitrary units)

|  | week | Ctrl | Lp | GT | GT+Lp |
| --- | --- | --- | --- | --- | --- |
| **PPARα** | 11 | 0.62 ± 0.12 | 0.87 ± 0.14 | 0.63 ± 0.08 | 0.76 ± 0.09 |
|  | **22** | 1.22 ± 0.15 *a* | 1.36 ± 0.09 *ab* | 0.83 ± 0.04 *b* | 1.04 ± 0.07 *ab* |
| PGC1α | 11 | 0.79 ± 0.10 | 0.63 ± 0.09 | 0.92 ± 0.05 | 1.03 ± 0.14 |
|  | 22 | 1.26 ± 0.12 | 1.32 ± 0.13 | 1.30 ± 0.11 | 1.01 ± 0.09 |
| **CD36** | **11** | 1.15 ± 0.20 *a* | 0.69 ± 0.07 *ab* | 0.58 ± 0.08 *b* | 0.48 ± 0.05 *b* |
|  | **22** | 0.70 ± 0.16 *a* | 1.20 ± 0.09 *b* | 0.74 ± 0.08 *a* | 0.77 ± 0.11 *ab* |
| ACADL | 11 |  |  |  |  |
|  | 22 | 0.76 ± 0.08 | 0.87 ± 0.06 | 0.90 ± 0.08 | 0.95 ± 0.10 |
| **LXR** | 11 | 1.74 ± 0.20 | 1.29 ± 0.20 | 1.37 ± 0.20 | 1.59 ± 0.20 |
|  | **22** | 1.50 ± 0.05 *a* | 1.47 ± 0.07 *ab* | 1.25 ± 0.14 *ab* | 1.09 ± 0.10 *b* |
| PXR | 11 |  |  |  |  |
|  | 22 | 1.07 ± 0.11 | 0.97 ± 0.04 | 1.10 ± 0.08 | 0.92 ± 0.08 |
| chREBP |  |  |  |  |  |
|  | 22 | 1.48 ± 0.12 | 1.50 ± 0.15 | 1.00 ± 0.15 | 1.17 ± 0.11 |
| XBP1 | 11 |  |  |  |  |
|  | 22 | 0.80 ± 0.05 | 0.76 ± 0.03 | 0.78 ± 0.04 | 0.94 ± 0.07 |
| PEPCK | 11 | 0,62 ± 0,08 | 0,66 ± 0,09 | 0,67 ± 0,05 | 0,79 ± 0,10 |
|  | 22 | 0.82 ± 0.13 | 0.87 ± 0.07 | 0.89 ± 0.07 | 0.96 ± 0.10 |
| CREB | 11 |  |  |  |  |
|  | 22 | 0.94 ± 0.07 | 0.99 ± 0.04 | 0.88 ± 0.07 | 1.06 ± 0.13 |
| GK |  |  |  |  |  |
|  | 22 | 0.72 ± 0.05 | 0.81 ± 0.06 | 0.63 ± 0.07 | 0.81 ± 0.08 |
| **SR-B1** |  |  |  |  |  |
|  | **22** | 0.60 ± 0.04 *a* | 0.74 ± 0.04 *ab* | 0.83 ± 0.04 *b* | 0.93 ± 0.04 *b* |
| **LDLR** |  |  |  |  |  |
|  | **22** | 1.12 ± 0.07 *a* | 1.01 ± 0.06 *ab* | 0.76 ± 0.05 *b* | 1.07 ± 0.06 *ab* |
| CYP7A1 |  |  |  |  |  |
|  | 22 | 0.69 ± 0.09 | 0.75 ± 0.09 | 0.85 ± 0.15 | 0.91 ± 0.15 |
| PAI-1 | 11 | 0.75 ± 0.11 | 0.72 ± 0.10 | 0.87 ± 0.16 | 0.53 ± 0.08 |
|  | 22 | 0.60 ± 0.08 | 1.03 ± 0.14 | 0.71 ± 0.19 | 0.79 ± 0.06 |
| **TLR4** | 11 | 0.98 ± 0.09 | 1.07 ± 0.11 | 0.95 ± 0.06 | 0.99 ± 0.05 |
|  | **22** | 0.77 ± 0.06 *ab* | 1.02 ± 0.09 *ab* | 0.71 ± 0.06 *a* | 1.08 ± 0.11 *b* |
| MyD88 | 11 |  |  |  |  |
|  | 22 | 0.99 ± 0.05 | 1.05 ± 0.05 | 1.04 ± 0.07 | 0.93 ± 0.06 |
| F4-80 |  |  |  |  |  |
|  | 22 | 0.66 ±0.08 | 0.70 ± 0.05 | 0.68 ± 0.12 | 0.76 ± 0.06 |

Values within a row not sharing the same letter indicate significant difference, p<0.05.

Bold indicate significant difference between groups.

For full names se additional file 5
